# Early diagnosis of Alzheimer’s Disease: Graph theoretical analysis of cerebellar network features based on ^18^F-AV45 PET

**DOI:** 10.1371/journal.pone.0342738

**Published:** 2026-02-17

**Authors:** Ruyi Li, Shaoping Jiang, Zhaoke Pi, Guisu Chen

**Affiliations:** 1 School of Mathematics and Computer Science, Yunnan Minzu University, Kunming, China; 2 National-Regional Key Technology Engineering Laboratory for Medical Ultrasound, Guangdong Key Laboratory for Biomedical Measurements and Ultrasound Imaging, School of Biomedical Engineering, Shenzhen University Medical School, Shenzhen University, Shenzhen, China; Banner Alzheimer's Institute, UNITED STATES OF AMERICA

## Abstract

Pathological and neuroimaging changes in the cerebellum of Alzheimer’s disease (AD) patients have been well documented. However, the changes in cerebellar amyloid plaque deposition connectivity networks during AD progression based on positron emission tomography (PET) imaging remain unclear. We selected ^18^F-florbetapir PET (^18^F-AV45 PET) imaging data from the Alzheimer’s disease neuroimaging initiative (ADNI) dataset (n = 612) and employed graph theoretical analysis to examine amyloid plaque deposition connectivity, comparing the connectivity differences across cognitively normal (CN), early mild cognitive impairment (EMCI), late mild cognitive impairment (LMCI), and AD groups. In addition, we combined graph theoretical features with the standardized uptake value ratio (SUVR) of regions of interest and applied them to machine learning models for the early diagnosis of AD. As cognitive decline progressed, significant changes in cerebellar network connectivity were observed across groups. Regarding local connectivity, changes in betweenness centrality were evident in multiple cerebellar regions at different cognitive stages. Cerebellar amyloid networks revealed early changes in amyloid plaque deposition connectivity. The machine learning model achieved an area under the curve (AUC) of 0.950 for distinguishing AD from CN, 0.995 for CN vs. EMCI, 0.964 for EMCI vs. LMCI and 0.632 for LMCI vs. AD. These findings provide new insights into the cerebellar pathological features of AD and highlight the potential of this approach for early identification and prediction of AD progression.

## Introduction

Alzheimer’s disease (AD) represents the most prevalent neurodegenerative condition and is the primary cause of dementia. It is marked by the gradual buildup of amyloid-beta (Aβ) plaques and tau neurofibrillary tangles, processes that begin decades before the appearance of dementia symptoms and progressively deteriorate over time. The progression of the disease may extend over a span of up to 30 years [1]. Identifying neurodegenerative changes at different stages, including cognitively normal (CN), early mild cognitive impairment (EMCI), late mild cognitive impairment (LMCI), and AD, is crucial for enabling timely therapeutic intervention.

Previous studies have consistently demonstrated that AD pathology predominantly involves selective neocortical layers and specific subcortical structures, particularly the parietal, temporal, frontal cortices, and hippocampus [2,3]. However, the role of the cerebellum in neurodegenerative diseases has often been underestimated [4]. The cerebellum plays a role in cognitive processes and emotional regulation, interacting with the cerebral cortex through a distinctive communication pathway [5]. Recent research has highlighted the crucial role of the cerebellum in cognitive functions, showing that damage to this area can result in cognitive impairments in patients with AD [6]. In addition, the cerebellum plays a key role in the onset and progression of AD [7]. In AD patients, the cerebellum exhibits pathological changes similar to those found in the cortical layers and hippocampus, such as Aβ deposition, p-Tau, and neuronal loss [8–10]. Consequently, the cerebellum has become a focal point in studying AD and other neurodegenerative disorders [11].

Graph theory is a mathematical tool for analyzing and quantifying brain networks [12]. By providing a simple model of the brain’s connectome, graph theory considers the entire network structure, represented by nodes and edges [13]. Electrophysiological data and graph theory analyses have revealed synaptic disruption and changes in functional connectivity in AD [14,15]. Topological metrics of AD patients hippocampal and medial temporal lobe structural networks have been uncovered [16]. Recent magnetic resonance imaging (MRI) studies have demonstrated the predictive value of both cerebellar radiological signatures and network characteristics in AD [17]. Graph theory allows for quantifying of brain structural network organization by representing these networks as graphs [18]. Despite extensive research based on graph theory to analyze AD’s pathological features and topological properties, studies on cerebellar topological networks based on positron emission tomography (PET) imaging remain relatively limited. PET can quantify pathological burdens in AD sensitive regions by measuring the standardized uptake value ratio (SUVR) of Aβ [19]. PET is the only technique capable of spatially resolving AD pathology in the living human brain. While structural MRI is a cost effective alternative for measuring neurodegeneration, it has lower specificity for AD pathology compared to PET [20]. The relationship between amyloid pathology as measured by ^18^F-florbetapir PET (^18^F-AV45 PET) and various cognitive functions has been explored, with the occipital and temporoparietal regions mediating these relationships [21].

Graph theory analysis provides a better understanding of the deposition or metabolic connectivity patterns between different brain regions. The use of graph theory to quantify the ^18^F-FDG PET brain covariance has been shown to be particularly relevant to AD, demonstrating a correlation with pathological progression [22]. Clinical assessments, cerebrospinal fluid analysis, and ^18^F-FDG PET imaging indicate that, particularly in the early stages of AD, hypo-connectivity and hyper-connectivity play distinct roles, with metabolism in hyper-connected regions acting as a mediator in the neurodegeneration of core pathological areas [23]. Graph theory analysis has shifted from traditional population-based network studies to individual-based approaches [24,25]. For each group, partial correlation coefficients of brain regions are calculated, controlling for age and gender, and an undirected weighted metabolic connectivity matrix is constructed based on brain nodes for population-level graph theory analysis [24]. Individual-specific metabolic networks are constructed by adding each patient to the reference group, calculating the differences between disturbed networks and the reference network, and applying Z-score transformation to the individual difference networks, followed by graph theory analysis to assess network features [25]. Individual-level studies contribute to a deeper understanding of patient heterogeneity. The transition from group-based studies to individualized graph theory research not only enhances diagnostic precision but also serves as a complement to the traditional group-based research.

In PET studies, the amyloid PET derivative ^18^F-AV45 PET can detect Aβ plaque deposition in the brain [26]. Previous PET studies have indicated the presence of amyloid plaque deposition in the cerebellum of CN carriers, and the pathological changes typically manifest approximately a decade prior to the clinical onset of symptoms in autosomal dominant Alzheimer’s disease [27]. This makes it valuable for early AD diagnosis, especially when patients exhibit mild cognitive impairment or remain asymptomatic, as it can detect abnormal Aβ deposition, thus providing a basis for early intervention.

**Fig 1:**
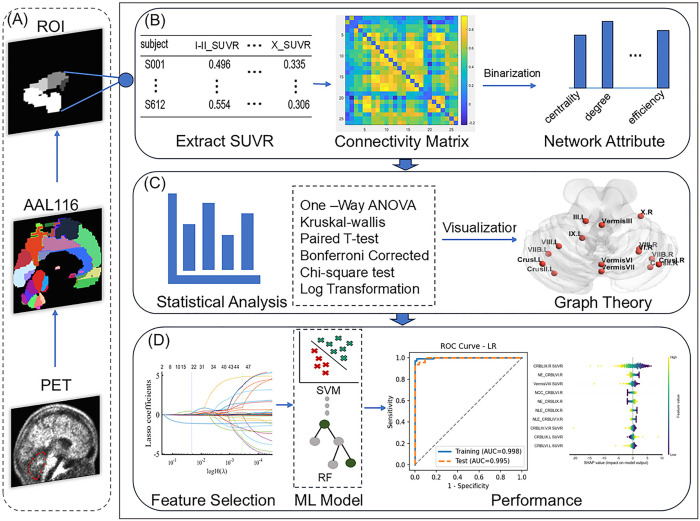
Workflow for Data Analysis Pipeline: **(A)** Data preprocessing steps. **(B)** Extraction of the SUVR for each cerebellar region, construction of connectivity matrices, and subsequent graph theory analysis. **(C)** Statistical analysis based on graph theory features and visualization of differential nodes. **(D)** Identification of the most relevant features through least absolute shrinkage and selection operator regression, using graph theory analysis features, with performance assessed via 5-fold cross-validation. The potential of cerebellar graph theory features for early prediction of AD was evaluated.

The primary objective of this investigation is to analyze changes in cerebellar amyloid plaque deposition connectivity across different cognitive states and apply these findings to early AD diagnostic classification (Fig 1). Specifically, we will compare differences between the CN and EMCI groups, EMCI and LMCI groups, and LMCI and AD groups. Using ^18^F-AV45 PET imaging data, we will employ graph theory analysis to construct a network model of the cerebellum and explore the impact of amyloid plaque deposition on the deposition connectivity of various cerebellar regions. The study will analyze changes in cerebellar network connectivity between groups, including metrics such as average degree, clustering coefficient (CC), and centrality, to reveal the characteristics of cerebellar connectivity changes across different cognitive states and their relationship to cognitive function. Furthermore, we hypothesize that features extracted from cerebellar graph theory analysis can provide insights into the potential pathology of AD. Therefore, our goal is to develop and evaluate machine learning (ML) models using cerebellar graph theory analysis features extracted from PET for the early diagnosis of AD.

## Materials and methods

### Subjects

The information utilized in this research was sourced from the Alzheimer’s disease neuroimaging initiative (ADNI) dataset (http://adni.loni.usc.edu). We accessed the ADNI database in June 2024. A total of 612 participants with corresponding MRI and PET imaging data were initially included. The data were obtained from three phases of the ADNI study: ADNI GO, ADNI 2, and ADNI 3. These participants comprised 172 CN individuals, 159 with EMCI, 141 with LMCI, and 140 with AD. All data were fully de-identified, and the authors had no access to any personally identifiable information during or after data collection. Detailed image acquisition is provided in the Supplementary Material S1 File. This analysis incorporated ^18^F-AV45 PET scan data and clinical variables, including demographic variables (age, sex) and cognitive assessment results (MMSE scores).

Ethical approval for ADNI was granted by the review boards of the involved institutions, and all participants provided informed consent before enrolling in the study.

### Data preprocessing

After acquiring the MRI and ^18^F-AV45 PET data, preprocessing of each subject’s brain images was performed using the SPM12 toolbox implemented in MATLAB. The procedures were as follows: first, all MRI and PET data were converted from DICOM to NIFTI format. PET images were then corrected for head motion to reduce artifacts caused by subject movement. Subsequently, the origins of the MRI and motion-corrected PET images were realigned to the anterior commissure posterior commissure line. Each PET image was then coregistered to its corresponding MRI image to achieve intermodal spatial alignment. The coregistered MRI images were segmented into gray matter, white matter, and cerebrospinal fluid, the spatial normalization was performed. Specifically, the coregistered MRI images were nonlinearly registered to the Montreal Neurological Institute (MNI) template, and the resulting deformation fields were applied to the corresponding PET images. The PET images were resampled to a voxel size of 2 × 2 × 2 mm³. Finally, partial volume effect correction (PVEc) of the spatially normalized PET images was performed using the geometric transfer matrix (GTM) method implemented in the PETPVE12 toolbox [28].

Then, intensity normalization of the PET brain images for all subjects was performed under both non-corrected and PVEc-GTM corrected conditions using the SNBPI Toolbox [29]. Specifically, Based on the AAL116 template, cerebellar and typical cortical brain region templates were created, with the typical cortical brain regions including areas of the brain with higher amyloid burden in AD, such as the frontal, temporal, parietal cortices, and precuneus [30]. The cerebellum was subdivided into 26 regions of interest, with the bilateral cerebellar lobules including Crus I, Crus II, III, IV, V, VI, VIIB, VIII, IX, XI, and the Vermis I, II, III, IV, V, VI, VII, VIII, IX, and X. Meanwhile, the frontal, temporal, parietal cortices, and precuneus were subdivided into 72 regions of interest (Supplementary Material Table S1 in S1 File). Using the updated_pons_vermis intensity normalization template provided in the SNBPI Toolbox, the pons was manually segmented in 3D Slicer (the version 5.8.1. link: https://slicer.Org. Supplementary material S1 File). The pons was used as the reference region to calculate the SUVRs for the 26 cerebellar subregions and 72 cortical regions under both non-corrected and PVEc-GTM corrected conditions [31]. The mean SUVRs for the 26 cerebellar regions in the CN, EMCI, LMCI, and AD groups were calculated under both PVEc and non-PVEc conditions (S2 and S3 Tables in S1 File). The formula for calculating the SUVR is as follows:


$$SUVR=\frac{{SUVR}_{ROI}}{{SUV}_{whole\_pons}}$$ (1)

The cerebellar and cortical SUVR_pons were calculated using this formula (1).

### Brain network construction

According to different data processing methods, We constructed individual-specific cerebellar and cortical deposition networks both with and without PVEc. In our parallel control study, a recently developed methodology was employed to construct individual-specific networks, thereby to obtain the individual-specific deposition network [25,32]. The construction process of individual-specific cerebellar deposition network without PVEc as follows: First, for n CN subjects, a group-level cerebellar deposition network was constructed using the covariance method, serving as the reference network (${REN}_{n}$). Specifically, a deposition network was constructed using all n CN subjects. The nodes in the network were defined as 26 regions of the cerebellum, and the edges between each pair of nodes represented the correlation coefficient of the SUVRs between brain regions. Then, we added k patients (EMCI, LMCI, AD) to the reference group, and a new cerebellar deposition network (${\text{CDN}}_{\text{n}+\text{1}}$) was constructed using n+1 subjects (n  reference subjects and 1 patient). Next, the difference between the perturbed network (${\text{CDN}}_{\text{n}+\text{1}}$) and the reference network (${\text{REN}}_{\text{n}}$) was calculated as ${\Delta \text{CDN}}_{\text{n}}={\text{CDN}}_{\text{n}+\text{1}}-{\text{REN}}_{\text{n}}$. Since ${\Delta \text{CDN}}_{\text{n}}\;$was shown to follow a symmetric distribution similar to a normal distribution, referred to as the “volcanic distribution,” a Z-score transformation was applied to ${\Delta \text{CDN}}_{\text{n }}$ as follows [33]:


$$z=\frac{{\Delta CDN}_{n}}{\frac{\text{1}-{CDN}_{n}^{\text{2}}}{n-\text{1}}}$$ (2)

where n=172  is the number of subjects in the reference group. In the individual-specific cerebellar deposition network of each patient, the weight of each edge was the Z-score obtained from the above Equation (2). Additionally, Fisher’s Z transformation was performed to ensure normality. Similarly, CN subjects were added one by one to the reference group to obtain the individual-specific cerebellar deposition network for healthy controls.

To further analyze, the cerebellar deposition network matrix was converted into a binary matrix, considering only positive correlations. A sparsity threshold ranging from 0.05 (the minimum sparsity threshold estimated using the ‘gretna_get_rmax.m’ code in Gretna software) to 0.35 with a step size of 0.01, was applied to exclude spurious connections and ensure network integrity [34]. Subsequently, the Gretna toolbox was used to calculate ten network properties for the cerebellar networks [35]. These measures encompassed global characteristics such as small-world metrics (sigma, lambda, and gamma), CC, characteristic path length, global efficiency, and local efficiency. Additionally, nodal characteristics were considered, including degree centrality, betweenness centrality (BC), nodal CC, nodal efficiency, and nodal local efficiency. While global measures reflect the overall structure and functionality of the network, nodal measures pertain to specific cerebellar lobules within the network. Notably, BC refers to the number of shortest paths that pass through a given node in the network, serving as an indicator of the node’s significance and influence within the overall network structure.

Similarly, individual-specific cerebellar and cortical deposition networks with PVEc correction were constructed according to the above method (S4 and S6 Table in S1 File).

### Statistical analyses

Statistical processing was implemented through SPSS 27.0 (IBM Corp) and MATLAB R2021a. Chi-square testing was employed to examine intergroup variations in categorical measures. The normality of continuous demographic and graph-theoretic variables was verified using Shapiro-Wilk tests. The selection between independent *t*-tests and Wilcoxon rank-sum tests for intergroup analysis was determined by normality test results. The Bonferroni correction was applied for multiple comparisons across regions of interest (ROIs). The number of comparisons was determined separately for each brain area: 26 cerebellar ROIs and 72 cortical ROIs. Therefore, the corrected significance thresholds were set to p < 0.05/ 26 for the cerebellar analyses and p < 0.05/ 72 for the cortical analyses. Network characteristics exhibiting non-normal distributions underwent log-transformation to meet normality assumptions for comparative analyses. Demographic variables (age and gender) were controlled as covariates in all analyses, with intergroup differences evaluated using two-tailed Student’s t-tests. Statistical significance was defined as achieving a *p*-value below the 0.05 threshold.

### Model development, evaluation and interpretation

The classification features were extracted from individual data, including the region of interest SUVR_pons and individual-specific deposition network graph theory features, which encompassed both global and local attributes. To identify statistically significant features, t-tests and Mann- Whitney U tests were performed. Subsequently, the Least absolute shrinkage and selection operator (LASSO) was employed to identify the key graph theory features across the different groups. LASSO enhances model accuracy by shrinking the coefficients of less relevant features to zero, thereby improving feature selection. The optimal λ value was determined through 5-fold cross-validation by minimizing the validation error.

In this study, stratified random sampling was used to divide the dataset into two groups: a training set (containing 70% of the total data) and a test set (containing the remaining 30%). To further assess the model’s performance, six machine learning classifiers were employed: Support Vector Machine (SVM), Random Forest (RF), Logistic Regression (LR), Multilayer Perceptron (MLP), eXtreme Gradient Boosting (XGBoost), and k-Nearest Neighbors (k-NN). To identify the optimal parameters for these six models, a 5-fold cross-validation was performed for hyperparameter tuning. To ensure the validity of evaluation, the test set was not used during the model tuning phase. The specific parameters for each model are provided in S8 Table in S1 File. For a comprehensive evaluation of classification performance, several metrics were employed, including accuracy, specificity, sensitivity, Area Under the Curve (AUC), and Receiver Operating Characteristic (ROC) curves, and their corresponding 95% confidence intervals (CI). Only the model with the best classification results across different groups is presented in this study, while the performance of other models can be found in S9 Table in S1 File. The source code for this method will be freely available at https://github.com/LYRLJL/cerebellum_machine-learning.

### Model interpretability with shapley additive explan

The Shapley additive explanations (SHAP) framework was used to quantify the feature-specific contributions of cerebellar network components and the SUVR of different cerebellar regions to model prediction accuracy. This approach, based on Shapley values derived from cooperative game theory, allows us to quantify the contribution of each feature to the predictions of the most effective model. Shapley values provide a measure of the importance of each feature in enhancing classification performance, and they are applicable to various feature types. The SHAP feature importance plot ranks attributes along the vertical axis by their contribution magnitude, with a chromatic scale (yellow denoting maximal values, purple indicating minimal values) encoding feature-value relationships.

## Results

### Demographic and clinical comparisons

Table 1 provides an overview of the demographic characteristics of all participants. The study sample included 172 individuals with CN status (98 males, 74 females; mean age 75.89 ± 7.97 years), 159 patients diagnosed with EMCI (78 males, 81 females; mean age 72.99 ± 7.04 years), 141 patients with LMCI (67 males, 74 females; mean age 72.75 ± 7.37 years), and 140 patients with AD (82 males, 58 females; mean age 74.41 ± 8.40 years). MMSE scores showed statistically significant variations among the study groups (p < 0.05). Significant differences in the mean values of PVEc, non-PVEc cerebellum, and mean cortical SUVR were observed across all group comparisons (CN vs. AD, CN vs. EMCI, EMCI vs. LMCI, LMCI vs. AD), except between the EMCI and LMCI groups.

**Table 1:** Presents the baseline demographic and clinical features of study participants.

Characteristic	CN (n = 172)	EMCI (n = 159)	*p*-value
Age	75.89 ± 7.97	72.99 ± 7.04	0.001^a^
MMSE	28.77 ± 1.67	27.8 ± 4.26	<0.001^a^
Gender(Male/Female)	74/98	81/78	0.150^b^
Mean cerebellar SUVR	0.55(0.52,0.60)	0.58(0.55,0.62)	<0.001^c^
Mean cerebellar SUVR_PVEc	0.48 (0.45, 0.51)	0.50 (0.47, 0.54)	<0.001^c^
Mean cortical SUVR	0.62(0.57,0.71)	0.70(0.63,0.86)	<0.001^c^
	**EMCI (n = 159)**	**LMCI (n = 141)**	***p*-value**
Age	72.99 ± 7.04	72.75 ± 7.37	0.777^a^
MMSE	27.8 ± 4.26	25.58 ± 3.56	<0.001^a^
Gender(Male/Female)	81/78	74/67	0.791^b^
Mean cerebellar SUVR	0.58(0.55,0.62)	0.60(0.56,0.63)	0.113^c^
Mean cerebellar SUVR_PVEc	0.50 (0.47, 0.54)	0.51(0.49,0.55)	0.223
Mean cortical SUVR	0.70(0.63,0.86)	0.78(0.66,0.88)	0.051 ^c^
	**LMCI (n = 141)**	**AD (n = 140)**	***p*-value**
Age	72.75 ± 7.37	74.41 ± 8.40	0.081^a^
MMSE	25.58 ± 3.56	21.45 ± 4.26	<0.001^a^
Gender(Male/Female)	74/67	58/82	0.306^b^
Mean cerebellar SUVR	0.60(0.56,0.63)	0.63(0.59,0.68)	<0.001^c^
Mean cerebellar SUVR_PVEc	0.51(0.49,0.55)	0.54 (0.51,0.59)	<0.001^c^
Mean cortical SUVR	0.78(0.66,0.88)	0.88(0.80,0.96)	<0.001^c^
	**CN (n = 172)**	**AD (n = 140)**	***p*-value**
Age	75.89 ± 7.97	74.41 ± 8.40	0.111^a^
MMSE	28.77 ± 1.67	21.45 ± 4.26	0.001^a^
Gender(Male/Female)	74/98	58/82	0.301^b^
Mean cerebellar SUVR	0.55 (0.52,0.60)	0.63 (0.59,0.68)	<0.001^c^
Mean cerebellar SUVR_PVEc	0.48 (0.45,0.51)	0.54 (0.51,0.59)	<0.001^c^
Mean cortical SUVR	0.62(0.57,0.71)	0.88(0.80,0.96)	<0.001^c^

**a**: T-test, **b**: Chi-square test, **c**: Mann-Whitney test.

### Changes in amyloid plaque deposition connectivity in CN to AD

**Fig 2:**
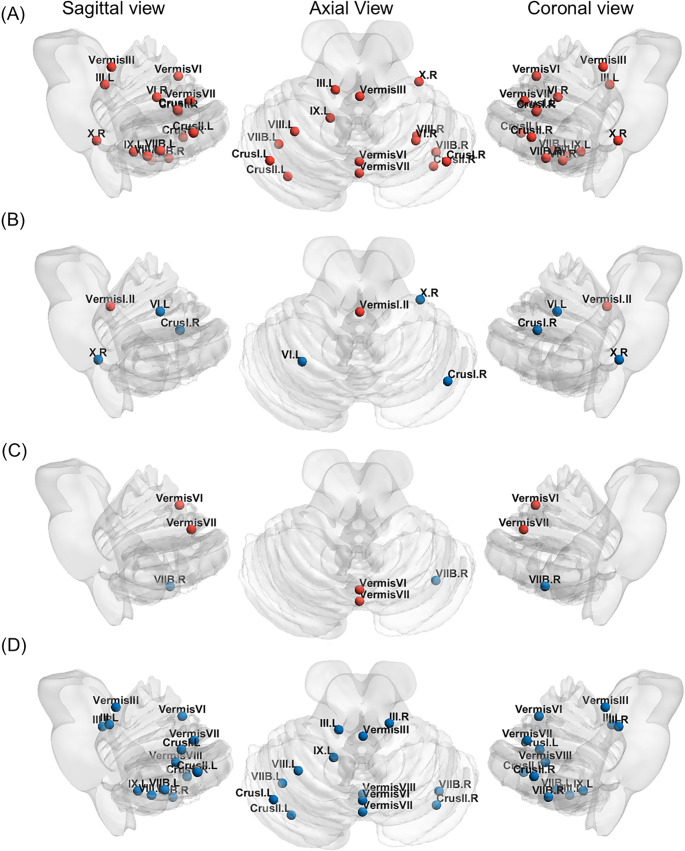
Cerebellar Graph Theory Analysis of CN, EMCI, LMCI, and AD Individuals. Cerebellar graph theory analysis reveals significant differences in amyloid plaque deposition connectivity between the **CN, EMCI**, LMCI, and **AD** groups: **(A)** AD vs. CN group; **(B)** CN vs. EMCI group; **(C)** EMCI vs. LMCI group; **(D)** LMCI vs. AD group in terms of significant differences in betweenness centrality (*p* < 0.05, Bonferroni correction). The nodes represent 26 cerebellar lobules, with red and blue indicating regions with significantly increased and decreased intermediary centrality. L,left; R, right.

**Fig 3:**
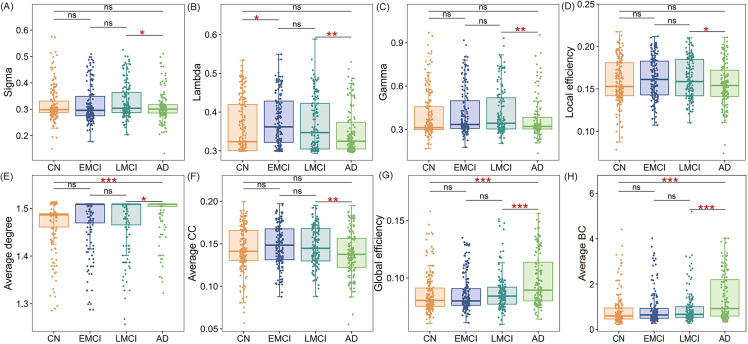
Inter-group comparison of cerebellar amyloid plaque deposition connectivity in the study groups. The data illustrate the distribution of changes in cerebellar network connectivity, including small-world properties, average degree, clustering coefficient, and centrality. *P*-values were obtained after correcting for multiple comparisons across the 26 cerebellar regions using false discovery rate methods (* *p* < 0.05; ** *p* < 0.01; *** *p* < 0.001). CN: cognitively normal, EMCI: early mild cognitive impairment, LMCI: late mild cognitive impairment, AD: Alzheimer’s disease, CC: clustering coefficient, BC: betweenness centrality.

Compared to the CN group, the AD group exhibited significant alterations in cerebellar connectivity (Table 2, Fig 3), specifically characterized by an increase in both average degree (1.463 vs. 1.489, *p* < 0.001; Table 2, Fig 3E) and global network efficiency (0.086 vs. 0.098, *p* < 0.001; Table 2, Fig 3G). Topological analysis revealed a significant increase in the cerebellar BC values in several regions when comparing AD patients with CN individuals. These regions included the left Crus I, Crus II, III, VIIB, VIII, IX, right Crus I, Crus II, VI, VIIB, VIII, X, as well as Vermis III, Vermis VI, and Vermis VII. (*p* < 0.05, Bonferroni corrected; Fig 2A). The cortical network metrics exhibited significant changes, with the small-world properties Sigma, Lambda, and Gamma values significantly decreased, while global network efficiency, local efficiency, average degree, and average CC significantly increased (S5 Table in S1 File). Moreover, significant changes in BC values were observed in multiple cortical regions (S1 Fig in S1 File).

**Table 2:** Comparison of amyloid accumulation connectivity alterations between CN, AD, EMCI, and LMCI.

	CN	AD	Difference	CI lower	CI upper	*p*-value^a^
Sigma	0.318	0.311	−0.007	−0.021	0.007	0.338
Lambda	0.361	0.348	−0.018	−0.027	0.002	0.089
Gamma	0.394	0.371	−0.023	−0.057	0.010	0.170
Local efficiency	0.158	0.156	−0.002	−0.008	0.003	0.365
Average degree	1.463	1.489	0.026	0.013	0.039	<0.001***
Average CC	0.144	0.139	−0.005	−0.011	0.001	0.080
Global efficiency	0.086	0.098	0.012	0.008	0.017	<0.001***
Average BC	0.866	1.402	0.536	0.334	0.737	<0.001***
	**CN**	**EMCI**	**Difference**	**CI lower**	**CI upper**	***p*-value**
Sigma	0.318	0.319	0.001	0.014	0.016	0.875
Lambda	0.361	0.380	0.019	0.005	0.034	0.011*
Gamma	0.394	0.417	0.023	−0.013	0.058	0.209
Local efficiency	0.158	0.163	0.005	−0.001	0.010	0.128
Average degree	1.463	1.471	0.008	−0.006	0.023	0.261
Average CC	0.144	0.148	0.004	−0.002	0.009	0.152
Global efficiency	0.086	0.087	0.001	−0.003	0.005	0.596
Average BC	0.866	0.946	0.080	−0.082	0.241	0.335
	**EMCI**	**LMCI**	**Difference**	**CI lower**	**CI upper**	***p*-value**
Sigma	0.319	0.330	0.011	−0.005	0.027	0.173
Lambda	0.380	0.369	−0.011	−0.026	0.005	0.165
Gamma	0.417	0.418	0.001	−0.036	0.039	0.936
Local efficiency	0.163	0.162	−0.001	−0.006	0.005	0.955
Average degree	1.471	1.471	0	−0.016	0.016	0.982
Average CC	0.148	0.147	−0.001	−0.006	0.004	0.704
Global efficiency	0.087	0.088	0.001	−0.003	0.005	0.686
Average BC	0.946	0.933	−0.013	−0.183	0.158	0.883
	**LMCI**	**AD**	**Difference**	**CI lower**	**CI upper**	***p*-value**
Sigma	0.330	0.311	−0.019	−0.034	−0.004	0.014*
Lambda	0.369	0.348	−0.021	−0.036	−0.005	0.008**
Gamma	0.418	0.371	−0.048	−0.083	−0.012	0.009**
Local efficiency	0.162	0.156	−0.007	−0.012	−0.001	0.020*
Average degree	1.471	1.489	0.018	0.003	0.032	0.022*
Average CC	0.147	0.139	−0.008	−0.014	−0.002	0.006**
Global efficiency	0.088	0.098	0.010	0.006	0.015	<0.001***
Average BC	0.933	1.402	0.469	0.261	0.677	<0.001***

**a**: Stands for the result of the Mann-Whitney test, **CI**: 95% confidence interval, *: *p* < 0.05, **: *p <* 0.01, ***: *p* < 0.001.

### Changes in amyloid plaque deposition connectivity in CN to EMCI

Statistical analyses revealed a significant increase in the small world property lambda in EMCI compared to CN (0.361 vs. 0.380, p = 0.011; Table 2, Fig 3B). Regarding local connectivity, region-specific alterations in cerebellar BC were identified in EMCI individuals relative to CN controls. Specifically, the BC values in the Vermis I and II lobules were significantly elevated, while the BC values in the right Crus I, right X, and left VI lobules were significantly decreased (*p* < 0.05, Bonferroni corrected; Fig 2B). The cortical network metrics showed significant increases in local efficiency, global efficiency, average CC values (S5 Table in S1 File). Moreover, significant changes in BC values were observed in multiple cortical regions (S2 Fig in S1 File).

### Changes in amyloid plaque deposition connectivity in EMCI to LMCI

Compared to the EMCI group, there were no significant changes in the overall cerebellar deposition connectivity in the LMCI group. Additionally, in terms of local connectivity, the LMCI group exhibited BC changes in only a few regions. Specifically, the cerebellar BC value in the right VIIB lobule was significantly decreased, while the BC values in the Vermis VI and Vermis VII lobules were significantly increased. (*p* < 0.05, Bonferroni corrected; Fig 2C). The cortical network metrics showed significant decreases in the small world property parameters (Lambda and Gamma values), while global efficiency values exhibited a significant increase (S5 Table in S1 File). Moreover, significant changes in BC values were observed in multiple cortical regions (S3 Fig in S1 File).

### Changes in amyloid plaque deposition connectivity in LMCI to AD

Compared to the LMCI group, the AD group exhibited significant changes in cerebellar connectivity (Table 2, Fig 3). Specifically, the small world properties were significantly altered, with a decrease in sigma (0.330 vs. 0.311, *p* = 0.014; Fig 3A), lambda (0.369 vs. 0.348, *p* = 0.008; Fig 3B), gamma (0.418 vs. 0.371, *p* = 0.009; Fig 3C), local efficiency (0.162 vs. 0.156, *p* = 0.020; Fig 3D), and average CC (0.147 vs. 0.139, *p* = 0.006; Fig 3F). Additionally, the average degree (1.471 vs. 1.489, *p* = 0.022; Fig 3E) and global efficiency (0.088 vs. 0.098, *p* < 0.001; Fig 3G) were significantly increased. In terms of local connectivity, the AD group demonstrated significant differences in BC across multiple cerebellar regions compared to the LMCI group. Specifically, BC was significantly decreased in the left Crus I, Crus II, III, VIIB, VIII, IX, the right Crus II, III, VIIB, and bilateral Vermis III, Vermis VI, Vermis VII, and Vermis VIII (*p* < 0.05, Bonferroni corrected; Fig 2D). For cortical network metrics, in the small world properties Sigma, Lambda, and Gamma showed significant decreases, while local efficiency, global efficiency, and average CC values exhibited significant increases (S5 Table in S1 File). Moreover, significant changes in BC values were observed in multiple cortical regions (S4 Fig in S1 File).

### Distinguishing performance of cerebellar network models and cortical brain models

**Fig 4:**
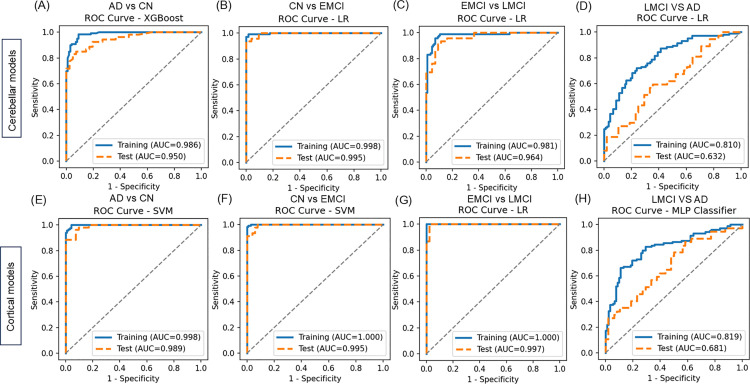
Performance of the cerebellum model and cortical brain model on the training and testing sets for binary classifications: CN vs. AD, CN vs. EMCI, EMCI vs. LMCI, and LMCI vs. AD.

In addition to the cerebellum model, a cortical brain model was construted by extracting cortical network feature and cortical SUVR features, Then the classification performance between the cerebellum and cortical models was compared, with their performance metrics presented in Table 3 and illustrated in Fig 4. The cerebellum model effectively distinguishes between AD and CN individuals, with XGBoost showing superior performance (S9 Table in S1 File). In the test group, the AUC was 0.950, accuracy was 0.862, sensitivity was 0.887, and specificity was 0.829. Similar results were observed in the training set (Table 3, Fig 4A). Furthermore, the cortical brain model outperformed the cerebellum model in classifying AD and CN, achieving an AUC of 0.989, accuracy of 0.926, sensitivity of 0.925, and specificity of 0.927 in the test set, with comparable performance in the training set (Table 3, Fig 4E). In the classification of CN vs. EMCI, EMCI vs. LMCI, and LMCI vs. AD, the LR classifier demonstrated stronger robustness in the cerebellar model. In the CN vs. EMCI test set, the AUC was 0.995, accuracy was 0.963, sensitivity was 0.936, and specificity was 0.981 (Table 3, Fig 4B). The cerebellum model performed better than the cortical model in distinguishing CN from EMCI (Table 3). In the EMCI vs. LMCI classification, the cortical brain model outperformed the cerebellum model. For the cerebellum model in the EMCI vs. LMCI classification, the AUC in the test set was 0.964, accuracy was 0.911, sensitivity was 0.913, and specificity was 0.909 (Table 3, Fig 4C). The cortical brain model, achieved an AUC of 0.997, accuracy of 0.956, sensitivity of 0.952, and specificity of 0.958 (Table 3, Fig 4G). In the LMCI vs. AD classification, the cerebellum model showed an AUC of 0.632, accuracy of 0.612, sensitivity of 0.698, and specificity of 0.523 in the test set. The cortical brain model showed similar performance to the cerebellum model (Table 3, Fig 4D, Fig 4H). These results suggest that the cerebellum model may have a comparative advantage in identifying MCI when compared with the cortical brain model.

**Table 3:** Classification performance of cerebellar graph-theoretical features for CN vs.AD, CN vs. EMCI, EMCI vs. LMCI, and LMCI vs. AD in the training and testing sets.

		Models	AUC	95% CI	Accuracy	95% CI	Sensitivity	95% CI	Specificity	95% CI	Classifier
AD vs CN	Cerebellar models	Training	0.987	(0.974,0.995)	0.922	(0.885,0.954)	0.916	(0.863, 0.963)	0.929	(0.875, 0.978)	XGBoost
		Testing	0.950	(0.904,0.983)	0.862	(0.787,0.926)	0.887	(0.796, 0.963)	0.829	(0.697, 0.936)	
	Cortical Model	Training	0.998	(0.996, 1.0)	0.973	(0.950,0.991)	0.975	(0.943, 1.0)	0.970	(0.933, 1.0)	SVM
			Testing	0.989	(0.973, 0.999)	0.926	(0.872,0.979)	0.925	(0.849, 0.983)	0.927	(0.842, 1.0)
CN vs EMCI	Cerebellar models	Training	0.998	(0.994, 1.0)	0.983	(0.965,0.996)	0.991	(0.971, 1.0)	0.975	(0.943, 1.0)	LR
		Testing	0.995	(0.986, 1.0)	0.963	(0.920, 0.990)	0.936	(0.860, 1.0)	0.981	(0.939, 1.0)	
	Cortical Model	Training	1.000	(0.999, 1.0)	0.991	(0.999, 1.0)	0.991	(0.967, 1.0)	0.991	(0.971, 1.0)	SVM
			Testing	0.995	(0.986, 1.0)	0.959	(0.918,0.990)	0.978	(0.925, 1.0)	0.941	(0.875, 1.0)
EMCI vs LMCI	Cerebellar models	Training	0.981	(0.963,0.995)	0.929	(0.891,0.962)	0.905	(0.840, 0.959)	0.948	(0.906,0.983)	LR
		Testing	0.964	(0.925, 0.991)	0.911	(0.844,0.967)	0.913	(0.822,0.980)	0.909	(0.821,0.981)	
	Cortical Model	Training	1.000	(0.998, 1.0)	0.981	(0.962,0.995)	0.990	(0.967, 1.0)	0.973	(0.940, 1.0)	LR
			Testing	0.997	(0.976, 1.0)	0.956	(0.9, 0.989)	0.952	(0.872, 1.0)	0.958	(0.891, 1.0)
LMCI vs AD	Cerebellar models	Training	0.810	(0.748, 0.865)	0.745	(0.684,0.806)	0.776	(0.685,0.853)	0.714	(0.627, 0.8)	LR
		Testing	0.632	(0.556,0.778)	0.612	(0.506,0.706)	0.698	(0.555,0.829)	0.523	(0.366,0.667)	
	Cortical Model	Training	0.819	(0.757,0.875)	0.755	(0.694,0.811)	0.721	(0.637, 0.800)	0.794	(0.705,0.874)	MLP
		Testing	0.681	(0.560,0.789)	0.624	(0.529,0.718)	0.487	(0.326,0.647)	0.729	(0.604,0.854)	

**Fig 5:**
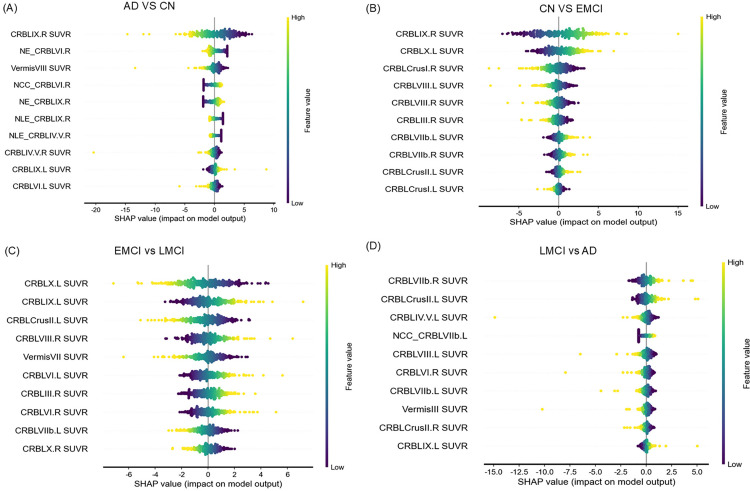
SHAP summary dot plots of the top 10 features of the best-performing ML models in different groups.

SHAP analysis was employed to evaluate, the contribution of individual features to the discriminative power of the cerebellar model. The results revealed that the SUVR values of cerebellar regions play a crucial role in distinguishing between different cognitive states. We found that several cerebellar region SUVR features emerged as key contributors to the statistical differentiation of AD, CN, EMCI, and LMCI (Fig 5). For example, SUVR of CRBLIX.L, CRBLVII.R, and CRBLCruslIL were important contributors in multiple classifications task. Specifically, in the AD vs CN comparison, cerebellar-specific deposition network features including NE_CRBLVI.R, NCC_CRBLVI.R, NE_CRBLIX.R, NLE_CRBLIX.R, and NLE_CRBLIV.V.R played a significant role in the classification process (Fig 5A). These finding indicate that the SUVR features of cerebellar regions play a vital role in identifying and differentiating between the various stages of cognitive impairment.

## Discussion

This study analyzed changes in cerebellar amyloid plaque deposition connectivity networks during the progression of AD using ^18^F-AV45 PET imaging. Notably, this is the first study to investigate cerebellar amyloid plaque deposition connectivity changes based on PET images. The results showed significant differences in cerebellar amyloid SUVR values across the progression of cognitive decline. Graph theory analysis was used to investigate, changes in cerebellar amyloid plaques across different cognitive stages. Compared with the CN group, AD patients showed a significant increase in average degree and global efficiency (1.463 vs. 1.489, 0.086 vs. 0.098). For the CN and EMCI groups, the small-world property index Lambda was also significantly higher in the EMCI group than in the CN group (0.361 vs. 0.380). In the comparison between the LMCI and AD groups, all graph-theoretical metrics showed significant differences. These findings reveal significant alteration in both global and local cerebellar network connectivity in AD patients. To improve early AD classification, we combined graph theory analysis features of cerebellar amyloid plaque deposition connectivity networks with SUVR values of various cerebellar regions. As shown in Table 3, we integrated both local and global features, further enhanced the diagnostic performance of early AD. These results also provide new evidence for the role of the cerebellum in early identification of AD during its preclinical stage.

Amyloid PET imaging faces challenges in detecting brainstem and cerebellar involvement due to previous studies that used the cerebellum or pons as reference regions, excluding their own involvement from detection [36]. However, increasing evidence suggests amyloid plaque deposition in the cerebellum during AD progression. While this finding provides background for our study, further investigation is needed. Recent studies have confirmed the presence of amyloid plaques in the cerebellum of AD patients, with amyloid deposition in the cerebellum being a common feature of early-onset AD [5]. Our findings align with these studies, by using the pons as a reference region, we observed significant differences in cerebellar SUVR between the EMCI and CN groups, suggesting that amyloid plaque deposition begins early in AD, consistent with AD’s pathological features. Consistent with the pathological features of the disease. Additionally, higher baseline SUVR values, typically based on amyloid plaque load using mean cortical SUVR, have been associated with greater cognitive decline [37]. Furthermore, amyloid pathology correlates negatively with various cognitive functions, with the occipital and temporal-parietal regions mediating these relationships [21]. Our research on cerebellar amyloid SUVR aligns with studies in the brain, where amyloid SUVR is elevated in the cerebellum, corresponding to cognitive decline, albeit not significantly in the EMCI to LMCI stages. The unique nature of the cerebellum suggests that it appears to be unaffected by the amyloid-related neuropathological burden in the medial temporal lobe; however, it may still be susceptible to neurodegenerative changes driven by inflammation, metabolism, or other cellular processes. Given these considerations, caution is advised when selecting the cerebellum as a “reference” region or when including it in imaging or analysis in neuroimaging and histopathological studies of dementia [38].

Aβ deposition is closely related to disease progression and the severity of cognitive symptoms and can be used to monitor disease progression [39]. However, the spatial variability of Aβ deposition limits quantitative analysis using SUVR-based PET imaging. Topological analysis of Aβ deposition patterns may more accurately reflect the progression of AD. In this study, we investigated the ^18^F-AV45 PET images of different stages of AD from a topological perspective at both individual and group levels. By measuring Aβ accumulation across various cerebellar regions and applying graph theory analysis, a comprehensive assessment of both global and local features was conducted. During the procession of AD, compared to CN, the mean values of degree centrality and global efficiency were significantly increased in AD. However, no significant changes in degree centrality, CC, local efficiency, and global efficiency were observed in the comparisons between CN and EMCI, as well as EMCI and LMCI. BC reflects the importance and influence of a node within the entire network, and these significant changes were particularly evident in the following cerebellar lobules: left Crus I, Crus II, III, VIIB, VIII, IX, right Crus II, III, VIIB, and bilateral Vermis III, Vermis VI, Vermis VII, and Vermis VIII. These observations confirm recent hypotheses regarding the involvement of different functional areas within the cerebellum, particularly highlighting the role of cerebellar regions in cognitive processes [11]. The Cerebellar cognitive-emotional syndrome arises following injury to the regions of the cerebellum responsible for cognition and emotion. It primarily affects the vestibular cerebellum located in lobule X, along with the adjacent regions of lobule IX, and involves lobules Crus I, Crus II, VI, VIIA, VIIB, and vermis VII [40]. Emerging evidence highlights the critical contribution of vermal cerebellar subregions to AD pathogenesis. Beyond its well-established roles in maintaining motor coordination and postural stability, the cerebellar vermis distinctly participates in affective modulation via its extensive neural connections with limbic circuitry. The vermis, particularly the lobules associated with emotional processing, is crucial for emotion-related memory, and disruption of vermal regions impairs global brain connectivity [41]. The anterior vermis could be a component of the sensorimotor representation system. Meanwhile, the posterior vermis is typically associated with emotional and cognitive functions [42]. Individuals with damage to the posterior vermis frequently display impairments in working memory, in addition to experiencing various cognitive and emotional disturbances [43]. Decreased volume or activity in the vermis is often seen in individuals with neuropsychiatric disorders, including major depressive disorder in adolescents, social memory deficits, and future gait impairments in Parkinson’s disease [41,44,45]. Our findings suggest that the cerebellar vermis may also play a significant role in the progression of AD. We observed early changes in cerebellar amyloid plaque deposition connectivity, particularly in regions I and II of the vermis, with significant changes consistent with MRI results [17]. Our findings suggest that cerebellar graph theoretical features may provide valuable insights into the unique characteristics of cerebellar involvement in MCI and AD patients.

The predictive value of the cerebellum for mild cognitive impairment may be higher than the changes observed in the cortical regions of the brain. Compared to other advanced methods (Table 4), our model, based on cerebellar amyloid plaque deposition connectivity networks, graph theory analysis features, and SUVR of cerebellar regions, demonstrated exceptional performance in diagnosing MCI using machine learning methods. Specifically, in early-stage classification, the AUC for CN vs. EMCI reached 0.995, with an accuracy of 0.963, showcasing performance that surpasses most existing algorithms. For the EMCI vs. LMCI classification, the accuracy reached 0.911, with an AUC of 0.964, outperforming current leading methods (Table 4). The single-brain region analysis in our study significantly reduced unstable variables associated with multi-brain region analysis, simplified the training process, and enhanced early-stage classification performance. However, the model’s performance in the CN vs. AD and LMCI vs. AD classifications still needs further improvement. We further utilized the SHAP framework to assess the contribution of individual features to the interpretability of the AD diagnostic model. Overall, during the diagnostic process, graph theory features, particularly cerebellar SUVR_pons, indirectly demonstrated that cerebellar features are more pronounced in staging diagnosis than visual assessments. Additionally, the extracted graph theory features hold significant value in predicting individual treatment outcomes.

**Table 4:** Comparative experiment results.

Study	Modality	Feature	Model	Task	ACC	Sensitivity	Specificity	AUC
2023 [47]	SMRI	texture features	SVM	CN(121)/AD(200)	0.838	0.685	0.927	0.920
2024 [48]	MRI,SNP, CAP	Pearson, Mutual information	FIL-DMGNN	CN(205)/AD (165)	0.932	0.933	0.915	0.931
				EMCI(186)/LMCI(163)	0.744	0.772	0.642	0.737
2024 [49]	MRI,PET,ASL	Radiomics features of the hippocampus region	LR	CN(53)/AD(51)	**0.967**	**100%**	**0.933**	0.980
2024 [50]	MRI	Radiomics features of the cerebellar gray matter and white matter	LightGBM	CN(146)/MCI(138)	0.746	0.776	0.757	0.776
2025 [51]	SMRI,SNP	Comprehensive Features	WTCNN	CN(200)/AD(200)	0.951	0.970	0.931	–
				CN(200)/MCI(280)	0.853	0.897	0.821	–
2025 [52]	MRI,PET	Multimodal Fusion	MMSDL	CN(263)/AD(218)	0.953	0.974	0.927	0.947
				CN(263)/MCI(475)	0.852	0.694	0.940	0.814
2025 [17]	MRI	Radiomics and morphological network features of the cerebellum	LR vLasso	CN(676)/AD(317)	0.786	0.807	0.768	0.854
			AdaBoost	MCI(634)/AD(317)	0.645	0.506	0.756	0.662
			AdaBoost	CN(676)/MCI(634)	0.792	0.738	0.849	0.864
**Ours**	**PET**	**Cerebellar Network Graph Theory Analysis Features**	XGBoost	CN(172)/AD(140)	0.862	0.887	0.829	0.950
			LR	CN(172)/EMCI(159)	**0.963**	**0.936**	**0.981**	**0.995**
			LR	EMCI(159)/LMCI(141)	**0.911**	**0.913**	**0.909**	**0.964**
			MLP	LMCI(141)/AD(140)	0.612	0.698	0.524	0.632

These results align with previous observations, indicating that the cerebellum is involved in cognitive deficits related to AD [4]. AD is typically characterized by cognitive impairment, and extensive research suggests that the cerebellum plays a critical role in generating and maintaining both cognitive and non-cognitive functions [46]. Almost the entire cerebellar region is involved in cognitive functions, and abnormalities in cerebellar structure and function can lead to impairments in both cognitive and non-cognitive functions. Cerebellar pathology in AD can be reliably detected and quantified using various imaging biomarkers, including MRI, PET, and electrophysiological measures. MRI studies have revealed that AD may alter both the structure and function of the cerebellum, with findings such as gray matter atrophy, volumetric changes, radiological features, and topological network disruptions observed across different stages of disease severity [17,46].

Prior studies have shown that electrophysiological changes in the cerebellum of AD-related APP/PS1 mice precede any pathological changes [53]. ^18^F-FDG PET studies have identified cerebellar metabolic dysfunction in AD, observing an increase in cerebellar metabolism during the transition from MCI to AD, with hypermetabolism detected specifically in the AD cerebellar regions [54]. The above studies underscore the critical role of the cerebellum in cognitive impairments associated with AD. These AD biomarkers vary in terms of accessibility, cost, invasiveness, and comprehensiveness. While they may represent the most reliable and information-rich methods, ^18^F-AV45 PET uniquely detects Aβ pathology within the cerebellum, providing crucial insights into the underlying disease mechanisms [55,56]. Pathological changes associated with AD can be detected even during the preclinical or EMCI stages, with graph theory-based analysis showing strong predictive performance. A more detailed quantitative assessment of connectivity changes in amyloid plaque deposition within individual cerebellar lobules is essential for understanding AD progression.

This study has several limitations. First, we used the pons as the reference region, while alternative reference regions may yield different results. Future studies should perform sensitivity analyses using results from different reference regions to assess robustness. Second, since ADNI is a multi-center dataset, site-related differences may influence graph-theoretical analyses and machine learning classification. Therefore, future work should validate the findings across multiple centers to ensure consistency and reliability. Third, future research will need to conduct additional validation analyses using external datasets. Fourth, the differences in the timing between MRI and PET scans may impact the study results. Future research will further consider this potential influence.

## Conclusion

In conclusion, using the ^18^F-AV45 PET imaging dataset, our findings highlight the presence of abnormal cerebellar amyloid plaque deposition connectivity networks in the early stages of AD. As cognitive impairment deepens and progresses into AD, alterations in the cerebellar amyloid plaque deposition connectivity network become increasingly severe, affecting both global and local cerebellar connectivity. These changes appear early on, which may aid in the early detection of AD.

## Supporting information

S1 File Supplementary Methods for Early Diagnosis of Alzheimer’s Disease: Graph Theoretical Analysis of Cerebellar Network Features Based on 18F-AV45 PET. (DOCX)
