# Supplementary material for: Early diagnosis of Alzheimer’s Disease: Graph theoretical analysis of cerebellar network features based on 18F-AV45 PET
Source: PLoS One. 2026 Feb 17;21(2):e0342738. doi: 10.1371/journal.pone.0342738 (PMC12912619; doi:10.1371/journal.pone.0342738)
Supplement: S1 File — (DOCX) [file pone.0342738.s001.docx]

**Supplementary Methods for Early Diagnosis of Alzheimer's Disease: Graph Theoretical Analysis of Cerebellar Network Features Based on 18F-AV45 PET**

**S 1:** **Data description**

The data used in this study were obtained from the Alzheimer’s Disease Neuroimaging Initiative (ADNI) database (http://adni.loni.usc.edu), which we accessed in June 2024. ADNI is a multi-center database; however, it is primarily organized by study phases, including ADNI 1 (2004–2010), ADNI GO (2009–2011), ADNI 2 (2011–2016), ADNI 3 (2016–2022), and ADNI 4 (2022–present). In this study, a total of 612 participants with both MRI and ^18^F-AV45 PET imaging data were initially included, drawn from three phases of ADNI: ADNI GO, ADNI 2, and ADNI 3. During these phases, MRI data were acquired on multiple 3T MRI scanners using scanner-specific T1-weighted sagittal 3D MPRAGE sequences. Meanwhile, ^18^F-AV45 PET data were acquired on multiple instruments with varying resolutions and according to platform-specific acquisition protocols. Detailed information on the imaging protocols employed across ADNI sites can be found on the ADNI website (http://adni.loni.usc.edu/methods/ ). Through the harmonization of different scanners and tracers, the ADNI PET Core has demonstrated the feasibility of conducting large-scale, multi-center PET studies[1].

**S 2: Pons**

The pons is the portion of the brainstem between the midbrain above and the medulla oblongata below[2]. Using the updated_pons_vermis intensity normalization template provided in the SNBPI Toolbox, the pons was manually segmented in 3D Slicer (the version 5.8.1. link: https:// slicer. org. Fig S1).

**
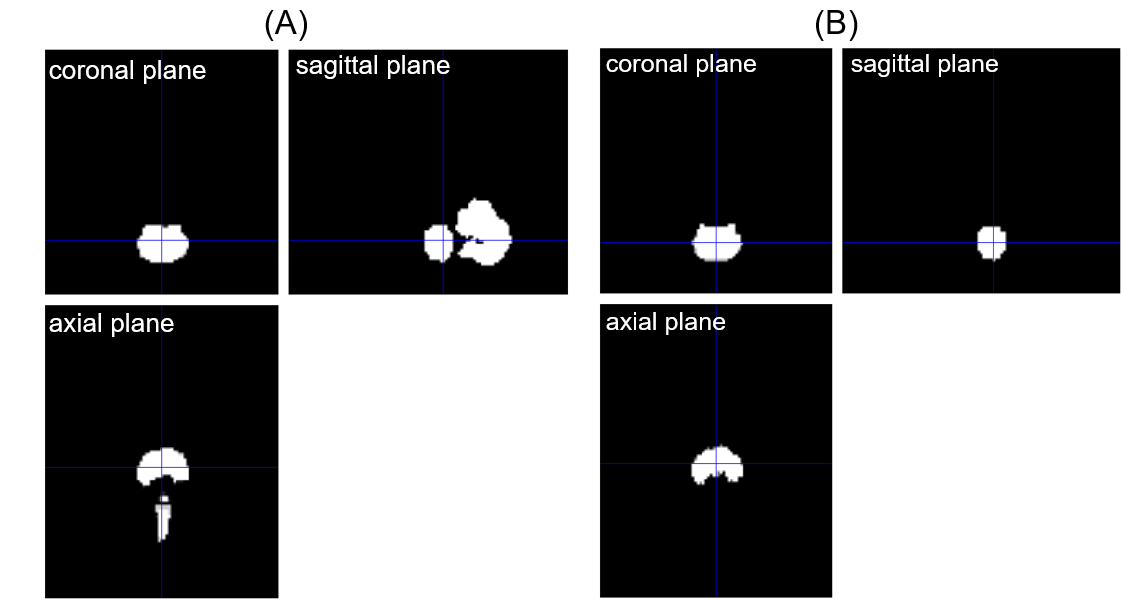
**

**Fig S1.** (A) updated_pons_vermis template. (B) Pons.

**S1 Table.** **Typical cortical regions with high amyloid burden in Alzheimer's disease selected based on the AAL116 template.**

| **ROI** |  |
| --- | --- |
| Precentral_L | 1 |
| Precentral_R | 2 |
| Frontal_Sup_L | 3 |
| Frontal_Sup_R | 4 |
| Frontal_Sup_Orb_L | 5 |
| Frontal_Sup_Orb_R | 6 |
| Frontal_Mid_L | 7 |
| Frontal_Mid_R | 8 |
| Frontal_Mid_Orb_L | 9 |
| Frontal_Mid_Orb_R | 10 |
| Frontal_Inf_Oper_L | 11 |
| Frontal_Inf_Oper_R | 12 |
| Frontal_Inf_Tri_L | 13 |
| Frontal_Inf_Tri_R | 14 |
| Frontal_Inf_Orb_L | 15 |
| Frontal_Inf_Orb_R | 16 |
| Rolandic_Oper_L | 17 |
| Rolandic_Oper_R | 18 |
| Supp_Motor_Area_L | 19 |
| Supp_Motor_Area_R | 20 |
| Olfactory_L | 21 |
| Olfactory_R | 22 |
| Frontal_Sup_Medial_L | 23 |
| Frontal_Sup_Medial_R | 24 |
| Frontal_Mid_Orb_L | 25 |
| Frontal_Mid_Orb_R | 26 |
| Rectus_L | 27 |
| Rectus_R | 28 |
| Insula_L | 29 |
| Insula_R | 30 |
| Cingulum_Ant_L | 31 |
| Cingulum_Ant_R | 32 |
| Cingulum_Mid_L | 33 |
| Cingulum_Mid_R | 34 |
| Cingulum_Post_L | 35 |
| Cingulum_Post_R | 36 |
| Hippocampus_L | 37 |
| Hippocampus_R | 38 |
| ParaHippocampal_L | 39 |
| ParaHippocampal_R | 40 |
| Amygdala_L | 41 |
| Amygdala_R | 42 |
| Cuneus_L | 43 |
| Cuneus_R | 44 |
| Fusiform_L | 45 |
| Fusiform_R | 46 |
| Postcentral_L | 47 |
| Postcentral_R | 48 |
| Parietal_Sup_L | 49 |
| Parietal_Sup_R | 50 |
| Parietal_Inf_L | 51 |
| Parietal_Inf_R | 52 |
| SupraMarginal_L | 53 |
| SupraMarginal_R | 54 |
| Angular_L | 55 |
| Angular_R | 56 |
| Precuneus_L | 57 |
| Precuneus_R | 58 |
| Paracentral_Lobule_L | 59 |
| Paracentral_Lobule_R | 60 |
| Heschl_L | 61 |
| Heschl_R | 62 |
| Temporal_Sup_L | 63 |
| Temporal_Sup_R | 64 |
| Temporal_Pole_Sup_L | 65 |
| Temporal_Pole_Sup_R | 66 |
| Temporal_Mid_L | 67 |
| Temporal_Mid_R | 68 |
| Temporal_Pole_Mid_L | 69 |
| Temporal_Pole_Mid_R | 70 |
| Temporal_Inf_L | 71 |
| Temporal_Inf_R | 72 |

**S2 Table. The standardized uptake values ratio of the non-PVEc cerebellar regions of interest using the ALL116 atlas (Nodes) in the CN, EMCI, LMCI, and AD groups.**

| Variables | AD (n = 140) | CN (n = 172) | EMCI (n = 159) | LMCI (n = 141) |
| --- | --- | --- | --- | --- |
|  |  |  |  |  |
| CrusI L | 0.54 ± 0.13 | 0.45 ± 0.08 | 0.49 ± 0.07 | 0.50 ± 0.08 |
| CrusI R | 0.53 ± 0.12 | 0.45 ± 0.08 | 0.47 ± 0.07 | 0.49 ± 0.07 |
| CrusII L | 0.51 ± 0.10 | 0.46 ± 0.09 | 0.51 ± 0.07 | 0.48 ± 0.07 |
| CrusII R | 0.47 ± 0.10 | 0.42 ± 0.08 | 0.46 ± 0.07 | 0.44 ± 0.07 |
| III L | 0.65 ± 0.18 | 0.60 ± 0.10 | 0.57 ± 0.06 | 0.60 ± 0.07 |
| III R | 0.63 ± 0.10 | 0.59 ± 0.09 | 0.54 ± 0.07 | 0.59 ± 0.07 |
| IV-V L | 0.60 ± 0.25 | 0.50 ± 0.08 | 0.50 ± 0.06 | 0.53 ± 0.07 |
| IV-V R | 0.63 ± 0.30 | 0.50 ± 0.08 | 0.50 ± 0.07 | 0.54 ± 0.07 |
| VI L | 0.66 ± 0.14 | 0.53 ± 0.08 | 0.54 ± 0.07 | 0.59 ± 0.09 |
| VI R | 0.65 ± 0.19 | 0.53 ± 0.08 | 0.54 ± 0.08 | 0.58 ± 0.08 |
| VIIB L | 0.54 ± 0.11 | 0.48 ± 0.07 | 0.53 ± 0.06 | 0.51 ± 0.06 |
| VIIB R | 0.53 ± 0.10 | 0.46 ± 0.08 | 0.51 ± 0.07 | 0.50 ± 0.07 |
| VIII L | 0.70 ± 0.11 | 0.63 ± 0.07 | 0.65 ± 0.06 | 0.66 ± 0.07 |
| VIII R | 0.68 ± 0.08 | 0.62 ± 0.07 | 0.64 ± 0.06 | 0.66 ± 0.07 |
| IX L | 0.71 ± 0.12 | 0.64 ± 0.06 | 0.65 ± 0.06 | 0.68 ± 0.06 |
| IX R | 0.67 ± 0.08 | 0.53 ± 0.08 | 0.64 ± 0.05 | 0.64 ± 0.06 |
| X L | 0.45 ± 0.14 | 0.40 ± 0.08 | 0.57 ± 0.08 | 0.44 ± 0.07 |
| X R | 0.41 ± 0.09 | 0.36 ± 0.08 | 0.47 ± 0.07 | 0.40 ± 0.06 |
| Vermis I-II | 0.77 ± 0.66 | 0.68 ± 0.10 | 0.63 ± 0.07 | 0.67 ± 0.11 |
| Vermis III | 0.47 ± 0.21 | 0.40 ± 0.08 | 0.39 ± 0.06 | 0.40 ± 0.06 |
| Vermis IV-V | 0.53 ± 0.35 | 0.42 ± 0.07 | 0.42 ± 0.06 | 0.45 ± 0.07 |
| Vermis VI | 0.56 ± 0.28 | 0.45 ± 0.07 | 0.46 ± 0.07 | 0.48 ± 0.09 |
| Vermis VII | 0.56 ± 0.10 | 0.50 ± 0.09 | 0.54 ± 0.08 | 0.52 ± 0.09 |
| Vermis VIII | 0.78 ± 0.17 | 0.67 ± 0.08 | 0.68 ± 0.08 | 0.71 ± 0.10 |
| Vermis IX | 0.72 ± 0.17 | 0.61 ± 0.09 | 0.64 ± 0.08 | 0.66 ± 0.11 |
| Vermis X | 0.68 ± 0.13 | 0.61 ± 0.08 | 0.62 ± 0.06 | 0.63 ± 0.08 |

Mean ± SD

SD: standard deviation

**S3 Table. The standardized uptake values ratio of the PVEc cerebellar regions of interest using the ALL116 atlas (Nodes) in the CN, EMCI, LMCI, and AD groups.**

| Variables | AD (n = 140) | CN (n = 172) | EMCI (n = 159) | LMCI (n = 141) |
| --- | --- | --- | --- | --- |
|  |  |  |  |  |
| CrusI L | 0.54 ± 0.08 | 0.47 ± 0.06 | 0.50 ± 0.08 | 0.50 ± 0.06 |
| CrusI R | 0.55 ± 0.08 | 0.48 ± 0.05 | 0.51 ± 0.08 | 0.51 ± 0.06 |
| CrusII L | 0.55 ± 0.10 | 0.48 ± 0.06 | 0.52 ± 0.10 | 0.52 ± 0.07 |
| CrusII R | 0.49 ± 0.09 | 0.42 ± 0.06 | 0.46 ± 0.09 | 0.46 ± 0.06 |
| III L | 0.60 ± 0.06 | 0.55 ± 0.04 | 0.58 ± 0.06 | 0.57 ± 0.04 |
| III R | 0.43 ± 0.05 | 0.40 ± 0.04 | 0.41 ± 0.05 | 0.41 ± 0.04 |
| IV-V L | 0.46 ± 0.08 | 0.37 ± 0.06 | 0.42 ± 0.08 | 0.42 ± 0.06 |
| IV-V R | 0.49 ± 0.09 | 0.38 ± 0.06 | 0.43 ± 0.09 | 0.44 ± 0.06 |
| VI L | 0.60 ± 0.10 | 0.49 ± 0.07 | 0.54 ± 0.10 | 0.55 ± 0.08 |
| VI R | 0.55 ± 0.10 | 0.46 ± 0.07 | 0.50 ± 0.10 | 0.50 ± 0.07 |
| VIIB L | 0.53 ± 0.09 | 0.45 ± 0.06 | 0.49 ± 0.10 | 0.49 ± 0.07 |
| VIIB R | 0.50 ± 0.10 | 0.43 ± 0.06 | 0.46 ± 0.10 | 0.46 ± 0.07 |
| VIII L | 0.65 ± 0.09 | 0.58 ± 0.06 | 0.62 ± 0.09 | 0.61 ± 0.06 |
| VIII R | 0.65 ± 0.09 | 0.59 ± 0.06 | 0.62 ± 0.09 | 0.62 ± 0.06 |
| IX L | 0.66 ± 0.08 | 0.60 ± 0.06 | 0.63 ± 0.09 | 0.62 ± 0.06 |
| IX R | 0.67 ± 0.08 | 0.60 ± 0.06 | 0.64 ± 0.09 | 0.63 ± 0.06 |
| X L | 0.43 ± 0.05 | 0.39 ± 0.04 | 0.41 ± 0.05 | 0.41 ± 0.04 |
| X R | 0.37 ± 0.05 | 0.33 ± 0.04 | 0.35 ± 0.06 | 0.34 ± 0.04 |
| Vermis I-II | 0.68 ± 0.08 | 0.66 ± 0.05 | 0.69 ± 0.08 | 0.66 ± 0.05 |
| Vermis III | 0.45 ± 0.05 | 0.41 ± 0.04 | 0.43 ± 0.06 | 0.42 ± 0.04 |
| Vermis IV-V | 0.42 ± 0.07 | 0.36 ± 0.05 | 0.39 ± 0.07 | 0.39 ± 0.05 |
| Vermis VI | 0.48 ± 0.09 | 0.41 ± 0.06 | 0.45 ± 0.09 | 0.45 ± 0.06 |
| Vermis VII | 0.62 ± 0.11 | 0.53 ± 0.07 | 0.58 ± 0.12 | 0.57 ± 0.08 |
| Vermis VIII | 0.67 ± 0.11 | 0.59 ± 0.07 | 0.63 ± 0.11 | 0.62 ± 0.08 |
| Vermis IX | 0.64 ± 0.10 | 0.56 ± 0.07 | 0.61 ± 0.11 | 0.60 ± 0.07 |
| Vermis X | 0.52 ± 0.09 | 0.49 ± 0.06 | 0.52 ± 0.11 | 0.50 ± 0.07 |

Mean ± SD

SD: standard deviation

**S4 Table.** Cerebellar Graph Theory Analysis: Basic Features and Differential Analysis.

| Variables | CN  (n = 172) | EMCI  (n = 159) | LMCI  (n = 141) | AD  (n = 140) | *P***^†^** |  |
| --- | --- | --- | --- | --- | --- | --- |
|  |  |  |  |  |  |  |
| Sigma | 0.32 ± 0.06 | 0.32 ± 0.07 | 0.33 ± 0.07 | 0.31 ± 0.06 | 0.149 |  |
| Lambda | 0.36 ± 0.07 | 0.38 ± 0.07 | 0.37 ± 0.07 | 0.35 ± 0.06 | **<0.001** |  |
| Gamma | 0.39 ± 0.16 | 0.42 ± 0.17 | 0.42 ± 0.16 | 0.37 ± 0.14 | 0.056 |  |
| Local efficiency | 0.16 ± 0.03 | 0.16 ± 0.02 | 0.16 ± 0.02 | 0.16 ± 0.02 | 0.126 |  |
| Average degree | 1.46 ± 0.06 | 1.47 ± 0.07 | 1.47 ± 0.07 | 1.49 ± 0.05 | **<0.007** |  |
| Average CC | 0.14 ± 0.03 | 0.15 ± 0.02 | 0.15 ± 0.02 | 0.14 ± 0.03 | **0.023** |  |
| Global efficiency | 0.09 ± 0.02 | 0.09 ± 0.02 | 0.09 ± 0.02 | 0.10 ± 0.02 | **<0.001** |  |
| Average BC | 0.87 ± 0.72 | 0.95 ± 0.77 | 0.93 ± 0.73 | 1.40 ± 1.02 | **<0.001** |  |

The data are presented as mean ± standard deviation. The adjusted means, controlled for age, with gender as a covariate, are used for group comparisons. **†** conducted an analysis using ANCOVA, with age and gender as covariates, for the four groups (CN, EMCI, LMCI, AD). CN: Cognitively Normal, EMCI: Early Mild Cognitive Impairment, LMCI: Late Mild Cognitive Impairment, AD: Alzheimer's Disease, CC: clustering coefficient, BC: betweenness centrality.

**S5 Table Comparison of Changes in Cortical Amyloid Protein Accumulation Connectivity between the CN, AD, EMCI, and LMCI Groups.**

|  | **CN** | **AD** | **Difference** | **CI lower** | **CI upper** | ***p*-value^a^** |
| --- | --- | --- | --- | --- | --- | --- |
| Sigma | 0.305 | 0.287 | -0.018 | -0.026 | -0.010 | <0.001*** |
| Lambda | 0.343 | 0.315 | -0.028 | -0.040 | -0.028 | <0.001*** |
| Gamma | 0.358 | 0.302 | -0.056 | -0.078 | -0.033 | <0.001*** |
| Local efficiency | 0.169 | 0.192 | 0.023 | 0.017 | 0.030 | <0.001*** |
| Average degree | 4.186 | 4.246 | 0.060 | 0.030 | 0.090 | <0.001*** |
| Average CC | 0.151 | 0.166 | 0.015 | 0.008 | 0.021 | <0.001*** |
| Global efficiency | 0.090 | 0.130 | 0.040 | 0.034 | 0.047 | <0.001*** |
| Average BC | 2.604 | 6.125 | 3.521 | 2.949 | 4.094 | <0.001*** |
|  | **CN** | **EMCI** | **Difference** | **CI lower** | **CI upper** | ***p*-value** |
| Sigma | 0.305 | 0.305 | 0 | -0.010 | 0.010 | 0.953 |
| Lambda | 0.343 | 0.347 | 0.004 | -0.010 | 0.019 | 0.534 |
| Gamma | 0.358 | 0.360 | 0.002 | -0.026 | 0.031 | 0.877 |
| Local efficiency | 0.169 | 0.180 | 0.011 | 0.006 | 0.017 | <0.001*** |
| Average degree | 4.186 | 4.187 | 0.001 | -0.032 | 0.038 | 0.944 |
| Average CC | 0.151 | 0.159 | 0.008 | 0.002 | 0.012 | 0.005** |
| Global efficiency | 0.090 | 0.101 | 0.011 | 0.005 | 0.017 | <0.001*** |
| Average BC | 2.604 | 3.598 | 0.994 | 0.460 | 1.529 | <0.001*** |
|  | **EMCI** | **LMCI** | **Difference** | **CI lower** | **CI upper** | ***p*-value** |
| Sigma | 0.305 | 0.296 | -0.009 | -0.017 | 0 | 0.054 |
| Lambda | 0.347 | 0.330 | -0.017 | -0.032 | -0.004 | 0.013* |
| Gamma | 0.360 | 0.331 | -0.029 | -0.055 | -0.003 | 0.030* |
| Local efficiency | 0.180 | 0.178 | -0.002 | -0.009 | 0.004 | 0.495 |
| Average degree | 4.187 | 4.207 | 0.020 | -0.018 | 0.057 | 0.305 |
| Average CC | 0.159 | 0.155 | -0.004 | -0.010 | 0.002 | 0.212 |
| Global efficiency | 0.101 | 0.109 | 0.008 | 0.001 | 0.016 | 0.027* |
| Average BC | 3.598 | 4.446 | 0.848 | 0.184 | 1.512 | 0.013* |
|  | **LMCI** | **AD** | **Difference** | **CI lower** | **CI upper** | ***p*-value** |
| Sigma | 0.296 | 0.287 | -0.009 | -0.016 | -0.002 | 0.012* |
| Lambda | 0.330 | 0.315 | -0.015 | -0.026 | -0.004 | 0.009** |
| Gamma | 0.331 | 0.302 | -0.029 | -0.049 | -0.008 | 0.006** |
| Local efficiency | 0.178 | 0.192 | 0.014 | 0.007 | 0.022 | <0.001*** |
| Average degree | 4.207 | 4.246 | 0.039 | 0.008 | 0.070 | 0.013 |
| Average CC | 0.155 | 0.166 | 0.011 | 0.004 | 0.018 | 0.003** |
| Global efficiency | 0.109 | 0.130 | 0.021 | 0.013 | 0.029 | <0.001*** |
| Average BC | 4.446 | 6.125 | 1.679 | 0.985 | 2.373 | <0.001*** |

**a**: Stands for the result of the Mann-Whitney test, **CI**: 95% confidence interval ,*****: *p* < 0.05, **:*p <* 0.01,****p*<0.001.

**S6 Table Comparison of PVEc cerebellar amyloid accumulation connectivity alterations between CN, AD, EMCI, and LMCI groups.**

|  | **CN** | **AD** | **Difference** | **CI lower** | **CI upper** | ***p*-value^a^** |
| --- | --- | --- | --- | --- | --- | --- |
| Sigma | 0.364 | 0.338 | -0.026 | -0.062 | 0.009 | 0.138 |
| Lambda | 0.356 | 0.342 | -0.014 | -0.035 | 0.007 | 0.188 |
| Gamma | 0.468 | 0.410 | -0.058 | -0.138 | 0.023 | 0.158 |
| Local efficiency | 0.162 | 0.158 | -0.004 | -0.012 | 0.003 | 0.232 |
| Average degree | 1.379 | 1.425 | 0.046 | -0.005 | 0.097 | 0.077 |
| Average CC | 0.147 | 0.138 | -0.009 | -0.017 | -0.001 | 0.032* |
| Global efficiency | 0.107 | 0.113 | 0.006 | -0.002 | 0.015 | 0.132 |
| Average BC | 1.562 | 1.848 | 0.286 | 0.071 | 0.499 | 0.009** |
|  | **CN** | **EMCI** | **Difference** | **CI lower** | **CI upper** | ***p*-value** |
| Sigma | 0.364 | 0.330 | -0.034 | 0.004 | 0.060 | 0.026 |
| Lambda | 0.356 | 0.338 | -0.018 | 0 | 0.037 | 0.046* |
| Gamma | 0.468 | 0.386 | -0.082 | 0.013 | 0.144 | 0.019* |
| Local efficiency | 0.162 | 0.159 | -0.003 | -0.011 | 0.003 | 0.217 |
| Average degree | 1.379 | 1.411 | 0.032 | -0.014 | 0.080 | 0.169 |
| Average CC | 0.147 | 0.142 | -0.005 | -0.012 | 0.002 | 0.141 |
| Global efficiency | 0.107 | 0.113 | 0.006 | -0.002 | 0.014 | 0.122 |
| Average BC | 1.562 | 1.683 | 0.121 | -0.062 | 0.308 | 0.191 |
|  | **EMCI** | **LMCI** | **Difference** | **CI lower** | **CI upper** | ***p*-value** |
| Sigma | 0.330 | 0.351 | 0.021 | -0.010 | 0.053 | 0.180 |
| Lambda | 0.338 | 0.352 | 0.014 | -0.006 | 0.034 | 0.166 |
| Gamma | 0.386 | 0.448 | 0.062 | -0.018 | 0.142 | 0.127 |
| Local efficiency | 0.159 | 0.159 | 0 | -0.006 | 0.008 | 0.801 |
| Average degree | 1.411 | 1.384 | -0.027 | -0.078 | 0.024 | 0.298 |
| Average CC | 0.142 | 0.147 | 0.005 | -0.007 | 0.008 | 0.887 |
| Global efficiency | 0.113 | 0.108 | -0.005 | -0.012 | 0.003 | 0.266 |
| Average BC | 1.683 | 1.612 | -0.071 | -0.259 | 0.116 | 0.454 |
|  | **LMCI** | **AD** | **Difference** | **CI lower** | **CI upper** | ***p*-value** |
| Sigma | 0.351 | 0.338 | -0.013 | -0.024 | 0.051 | 0.469 |
| Lambda | 0.352 | 0.342 | -0.010 | -0.013 | 0.032 | 0.405 |
| Gamma | 0.448 | 0.410 | -0.038 | -0.054 | 0.130 | 0.416 |
| Local efficiency | 0.159 | 0.158 | -0.001 | -0.010 | 0.006 | 0.645 |
| Average degree | 1.384 | 1.425 | 0.041 | -0.014 | 0.096 | 0.146 |
| Average CC | 0.147 | 0.138 | -0.009 | -0.013 | 0.004 | 0.286 |
| Global efficiency | 0.108 | 0.113 | 0.005 | -0.004 | 0.013 | 0.264 |
| Average BC | 1.612 | 1.848 | 0.236 | 0.018 | 0.453 | 0.034* |

**a**: Stands for the result of the Mann-Whitney test, **CI**: 95% confidence interval ,*****: *p* < 0.05, **:*p <* 0.01,****p*<0.001.

**
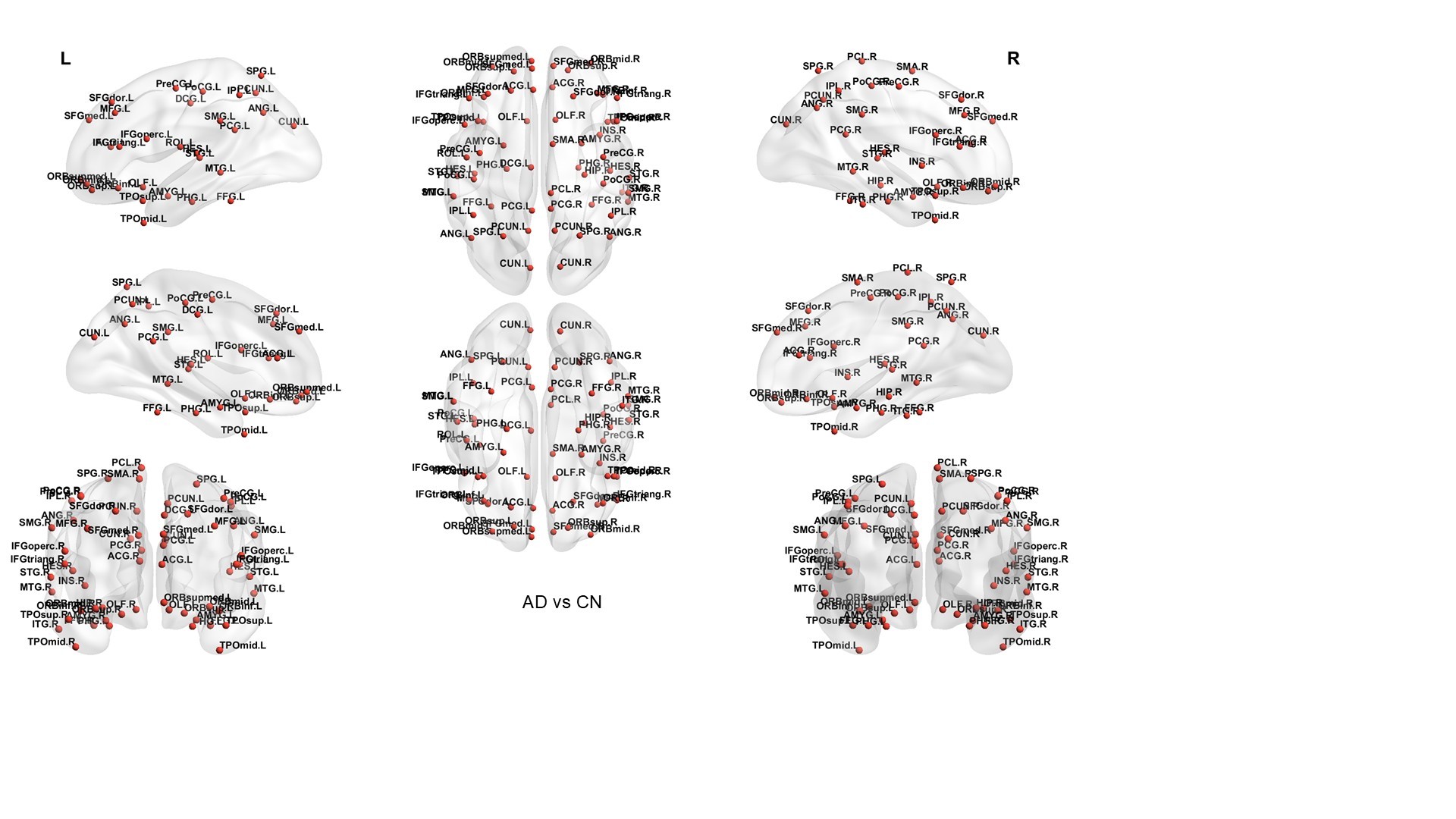
**

**Supplementary material Fig S1.** Cortical Graph Theory Analysis of CN and AD Groups. The cortical graph theory analysis reveals significant differences in amyloid plaque deposition connectivity between the CN and AD groups, manifested as significant differences in intermediary centrality (p < 0.05, Bonferroni correction). Nodes represent 72 cortical regions, with red and blue indicating regions with significantly increased and decreased intermediary centrality, respectively. L, left; R, right.

**
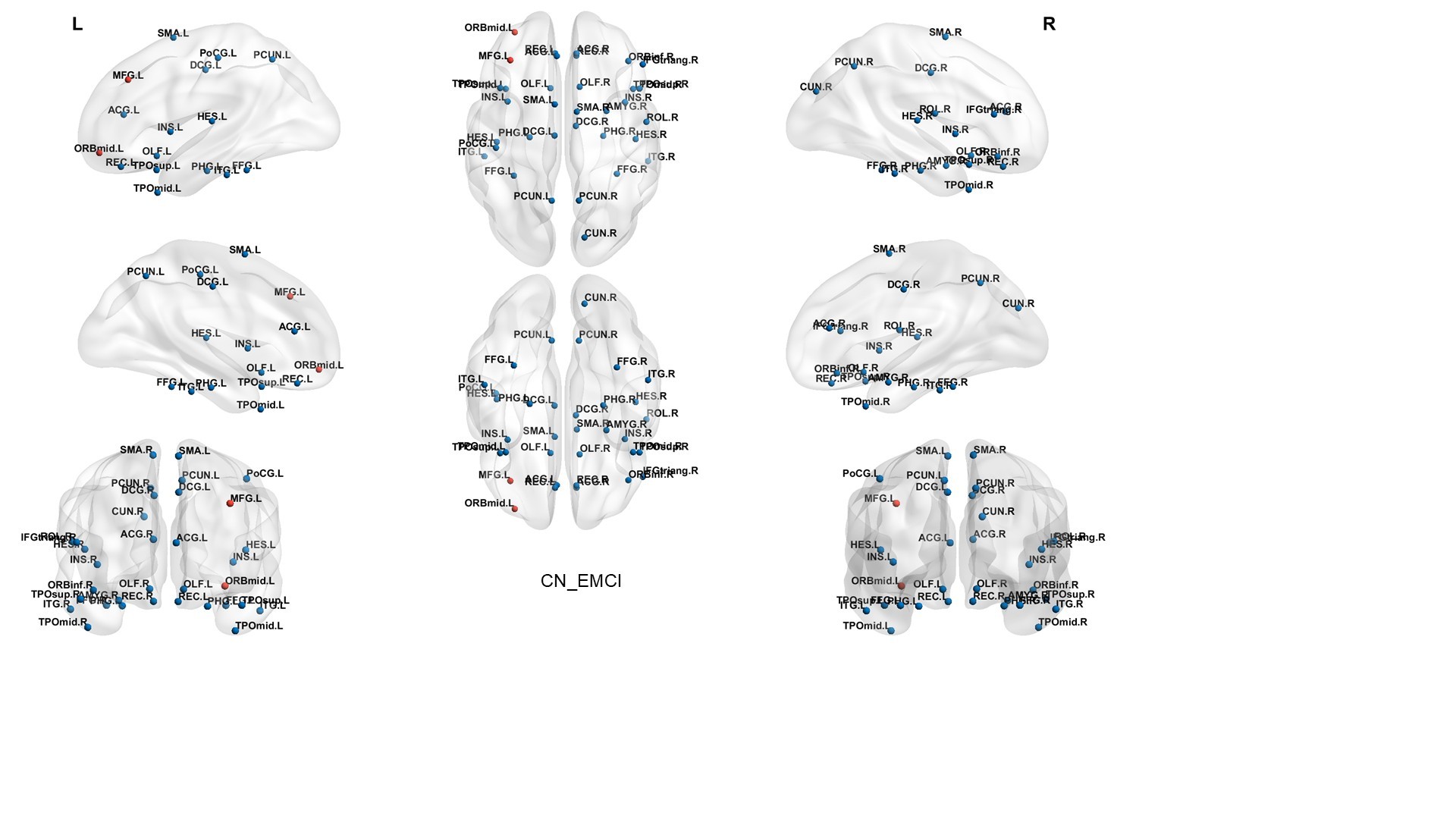
**

**Supplementary material Fig S2.** Cortical Graph Theory Analysis of CN and EMCI Groups. The cortical graph theory analysis reveals significant differences in amyloid plaque deposition connectivity between the CN and EMCI groups, manifested as significant differences in intermediary centrality (p < 0.05, Bonferroni correction). Nodes represent 72 cortical regions, with red and blue indicating regions with significantly increased and decreased intermediary centrality, respectively. L, left; R, right.

**
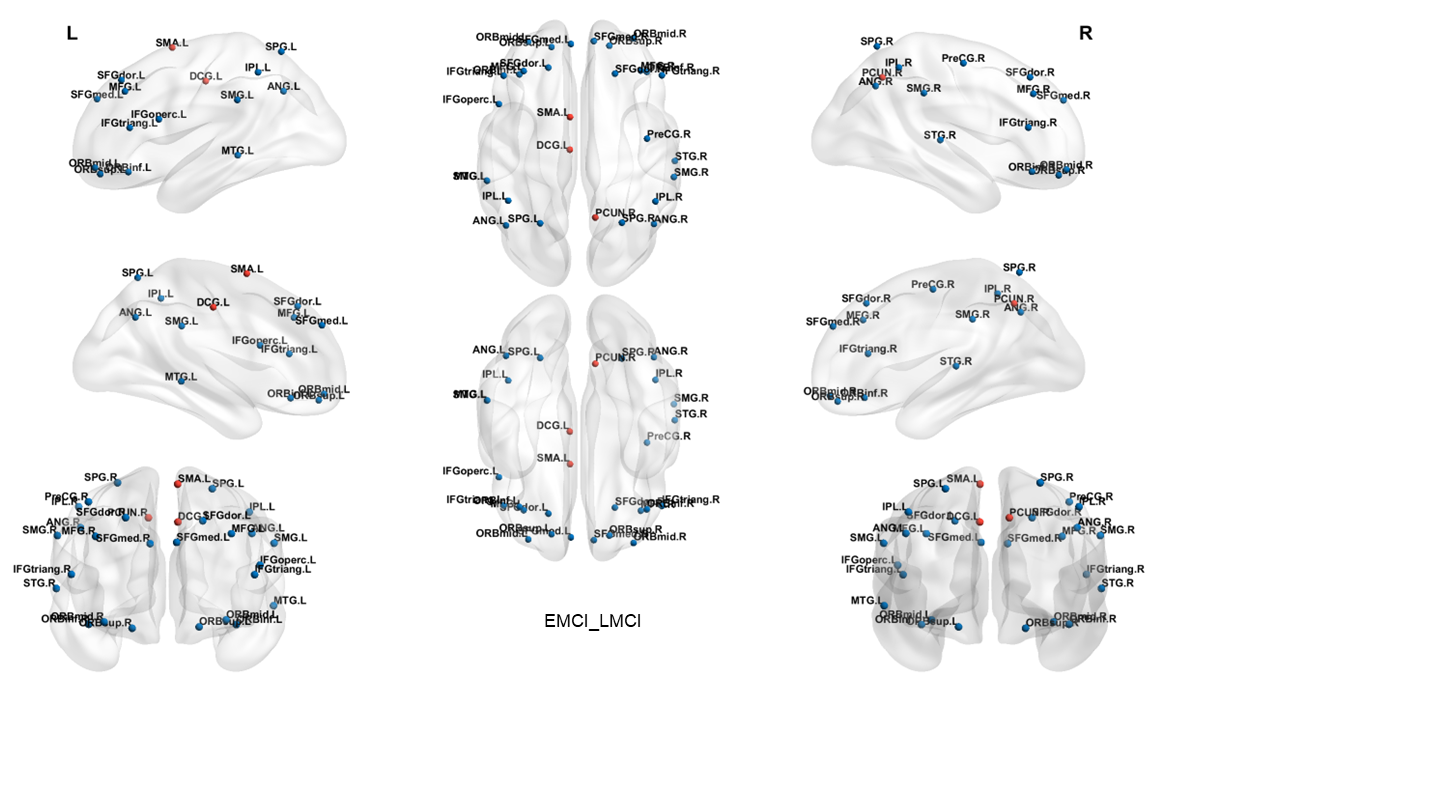
**

**Supplementary material Fig S3.** Cortical Graph Theory Analysis of EMCI and LMCI Groups. The cortical graph theory analysis reveals significant differences in amyloid plaque deposition connectivity between the EMCI and LMCI groups, manifested as significant differences in intermediary centrality (p < 0.05, Bonferroni correction). Nodes represent 72 cortical regions, with red and blue indicating regions with significantly increased and decreased intermediary centrality, respectively. L, left; R, right.

**
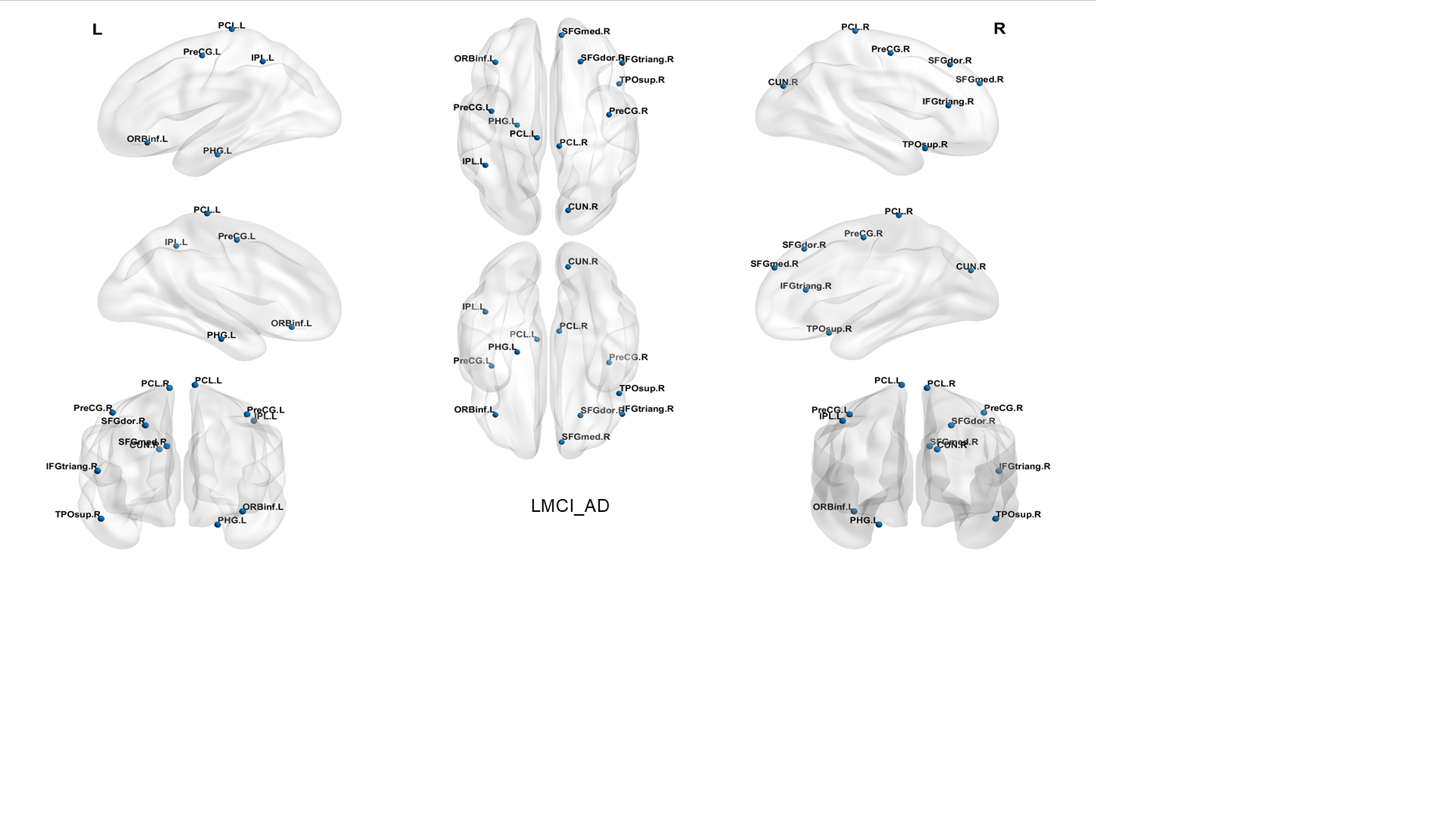
**

**Supplementary material Fig S4.** Cortical Graph Theory Analysis of LMCI and AD Groups. The cortical graph theory analysis reveals significant differences in amyloid plaque deposition connectivity between the LMCI and AD groups, manifested as significant differences in intermediary centrality (p < 0.05, Bonferroni correction). Nodes represent 72 cortical regions, with red and blue indicating regions with significantly increased and decreased intermediary centrality, respectively. L, left; R, right.

**S7 Table** Different Group Comparisons of Amyloid Plaque Connectivity Network Features (CN vs AD, CN vs EMCI, EMCI vs LMCI, LMCI vs AD).

| **CN vs AD** | | | | | | | | | | |
| --- | --- | --- | --- | --- | --- | --- | --- | --- | --- | --- |
| node | Degree Centrality | | Nodal Clust Coeff | | Nodal Efficiency | | Nodal Local Efficiency | | Betweenness centrality | |
|  | *t*-value | *p*-value | *t*-value | *p*-value | *t*-value | *p*-value | *t*-value | *p*-value | *t*-value | *p*-value |
| CrusI.L | 2.460 | 0.014 | 1.745 | 0.082 | **4.017** | **<0.001** | **2.069** | **0.039** | **3.034** | **0.003** |
| CrusI.R | 0.567 | 0.571 | -1.015 | 0.311 | 2.323 | 0.021 | -0.574 | 0.566 | **3.371** | **<0.001** |
| CrusII.L | 0.373 | 0.709 | -2.925 | 0.004 | 0.982 | 0.327 | -2.241 | **0.026** | **4.314** | **<0.001** |
| CrusII.R | -0.613 | 0.540 | -2.132 | 0.034 | 0.891 | 0.374 | -1.801 | 0.073 | **2.119** | **0.035** |
| III.L | -0.787 | 0.432 | -2.079 | 0.038 | 0.009 | 0.993 | -1.930 | 0.055 | **2.076** | **0.039** |
| III.R | -0.850 | 0.396 | -1.699 | 0.090 | 0.459 | 0.647 | -1.522 | 0.129 | 1.754 | 0.081 |
| IV V.L | 0.922 | 0.357 | 2.100 | 0.037 | **3.654** | **<0.001** | **2.379** | **0.018** | 1.197 | 0.232 |
| IV V.R | 2.632 | 0.009 | **3.600** | **<0.001** | **5.124** | **<0.001** | **3.865** | **<0.001** | 1.890 | 0.060 |
| VI.L | 1.182 | 0.238 | 2.652 | 0.008 | **4.086** | **<0.001** | **2.712** | **0.007** | 1.315 | 0.190 |
| VI.R | 1.698 | 0.091 | -0.155 | 0.877 | **3.496** | **<0.001** | 0.310 | 0.757 | **3.300** | **<0.001** |
| VIIB.L | -0.991 | 0.323 | **-3.843** | **<0.001** | 0.596 | 0.552 | **-3.208** | **0.002** | **4.508** | **<0.001** |
| VIIB.R | 0.772 | 0.441 | -1.508 | 0.133 | 1.760 | 0.079 | -0.892 | 0.373 | **4.080** | **<0.001** |
| VIII.L | 1.115 | 0.266 | -0.896 | 0.371 | 2.117 | 0.035 | -0.347 | 0.729 | **3.303** | **<0.001** |
| VIII.R | -0.043 | 0.966 | -0.885 | 0.377 | 2.150 | 0.032 | -0.675 | 0.500 | **3.698** | **<0.001** |
| IX.L | 1.615 | 0.107 | -0.013 | 0.990 | **3.237** | **0.001** | 0.450 | 0.653 | **1.991** | **0.047** |
| IX.R | 0.209 | 0.835 | 1.982 | 0.048 | 2.641 | 0.009 | **2.107** | **0.036** | 0.975 | 0.330 |
| X.L | -0.237 | 0.813 | -1.949 | 0.052 | 0.958 | 0.339 | -1.735 | 0.084 | 0.796 | 0.427 |
| X.R | 1.210 | 0.227 | -1.591 | 0.113 | 2.314 | 0.021 | -1.183 | 0.238 | **2.247** | **0.025** |
| Vermis I II | -0.316 | 0.753 | -1.310 | 0.191 | 0.248 | 0.804 | -1.109 | 0.268 | 1.488 | 0.138 |
| Vermis III | 0.459 | 0.646 | -0.786 | 0.433 | 1.476 | 0.141 | -0.460 | 0.646 | **2.615** | **0.009** |
| Vermis IV V | 0.778 | 0.437 | 1.150 | 0.251 | 2.966 | 0.003 | 1.433 | 0.153 | 1.935 | 0.054 |
| Vermis VI | -1.841 | 0.067 | -0.577 | 0.564 | 0.536 | 0.592 | -0.525 | 0.600 | **2.176** | **0.030** |
| Vermis VII | -1.229 | 0.220 | -2.265 | 0.024 | 0.180 | 0.858 | -1.913 | 0.057 | **2.787** | **0.006** |
| Vermis VIII | -0.703 | 0.482 | -0.550 | 0.583 | 1.412 | 0.159 | -0.607 | 0.544 | 1.544 | 0.124 |
| Vermis IX | -0.912 | 0.363 | -0.155 | 0.877 | 1.475 | 0.141 | -0.131 | 0.896 | 1.215 | 0.225 |
| Vermis X | -0.845 | 0.399 | -1.746 | 0.082 | 1.353 | 0.177 | -1.555 | 0.121 | 0.201 | 0.841 |
| **CN vs EMCI** | | | | | | | | | | |
| CrusI.L | -0.322 | 0.748 | -1.036 | 0.301 | -1.080 | 0.281 | -0.912 | 0.362 | -0.250 | 0.802 |
| CrusI.R | -0.460 | 0.646 | 1.010 | 0.313 | -0.683 | 0.495 | 0.753 | 0.452 | -2.251 | **0.025** |
| CrusII.L | -0.497 | 0.620 | 0.403 | 0.687 | -0.550 | 0.583 | 0.193 | 0.847 | -1.431 | 0.153 |
| CrusII.R | 0.064 | 0.949 | -0.195 | 0.845 | -0.329 | 0.742 | -0.139 | 0.889 | -0.030 | 0.976 |
| III.L | 0.596 | 0.552 | 0.125 | 0.900 | 1.292 | 0.197 | 0.319 | 0.750 | 1.001 | 0.317 |
| III.R | 1.144 | 0.253 | -0.434 | 0.665 | 1.429 | 0.154 | -0.112 | 0.911 | 1.455 | 0.147 |
| IV V.L | -0.399 | 0.691 | -1.247 | 0.213 | -0.671 | 0.503 | -1.153 | 0.250 | 0.151 | 0.880 |
| IV V.R | -2.831 | 0.005 | **-3.147** | **0.002** | -2.498 | 0.013 | **-3.226** | **0.001** | -0.543 | 0.588 |
| VI.L | -1.865 | 0.063 | -1.296 | 0.196 | -1.390 | 0.165 | -1.499 | 0.135 | **-2.373** | **0.018** |
| VI.R | -1.762 | 0.079 | -0.929 | 0.353 | -0.707 | 0.480 | -0.978 | 0.329 | -0.944 | 0.346 |
| VIIB.L | 0.588 | 0.557 | -0.307 | 0.759 | 0.165 | 0.869 | -0.309 | 0.758 | -0.606 | 0.545 |
| VIIB.R | 0.527 | 0.599 | -0.017 | 0.986 | 0.070 | 0.944 | 0.047 | 0.963 | -0.516 | 0.606 |
| VIII.L | 0.667 | 0.505 | 1.085 | 0.279 | 0.932 | 0.352 | 0.991 | 0.322 | -0.134 | 0.893 |
| VIII.R | 1.796 | 0.073 | 1.949 | 0.052 | 1.212 | 0.226 | 1.951 | 0.052 | -1.568 | 0.118 |
| IX.L | 0.594 | 0.553 | 1.906 | 0.058 | 0.386 | 0.700 | 1.596 | 0.111 | -0.561 | 0.575 |
| IX.R | 0.906 | 0.366 | **-3.342** | **<0.001** | 0.837 | 0.403 | **-3.210** | **0.002** | -0.402 | 0.688 |
| X.L | -0.705 | 0.482 | -2.048 | 0.041 | -1.252 | 0.211 | -1.702 | 0.090 | -0.672 | 0.502 |
| X.R | -2.149 | 0.032 | -1.338 | 0.182 | -2.264 | 0.024 | -1.633 | 0.103 | **-2.121** | **0.035** |
| Vermis I II | 2.300 | 0.022 | 1.289 | 0.198 | 2.404 | 0.017 | 1.563 | 0.119 | **2.840** | **0.005** |
| Vermis III | -0.877 | 0.381 | -0.618 | 0.537 | -0.548 | 0.584 | -0.480 | 0.632 | -1.076 | 0.283 |
| Vermis IV V | -0.835 | 0.404 | 0.055 | 0.957 | -0.671 | 0.503 | -0.218 | 0.827 | -1.764 | 0.079 |
| Vermis VI | 0.068 | 0.946 | 0.230 | 0.819 | -0.205 | 0.838 | 0.132 | 0.895 | -1.695 | 0.091 |
| Vermis VII | -0.529 | 0.597 | -0.410 | 0.682 | -1.069 | 0.286 | -0.584 | 0.560 | -1.585 | 0.114 |
| Vermis VIII | 1.157 | 0.248 | 0.966 | 0.335 | 0.480 | 0.631 | 1.034 | 0.302 | 0.292 | 0.771 |
| Vermis IX | 0.439 | 0.661 | 1.509 | 0.132 | 0.443 | 0.658 | 1.106 | 0.270 | -1.646 | 0.101 |
| Vermis X | 0.206 | 0.837 | 0.582 | 0.561 | -0.372 | 0.710 | 0.294 | 0.769 | 0.471 | 0.638 |
| **EMCI vs LMCI** | | | | | | | | | | |
| CrusI.L | -1.754 | 0.081 | -1.688 | 0.092 | -1.243 | 0.215 | -1.810 | 0.071 | 0.909 | 0.364 |
| CrusI.R | 0.436 | 0.663 | 0.628 | 0.530 | 0.455 | 0.650 | 0.588 | 0.557 | -0.080 | 0.937 |
| CrusII.L | 1.386 | 0.167 | 1.228 | 0.221 | 1.446 | 0.149 | 1.385 | 0.167 | 0.533 | 0.594 |
| CrusII.R | 0.619 | 0.537 | 0.849 | 0.396 | 0.709 | 0.479 | 0.762 | 0.447 | 0.603 | 0.547 |
| III.L | 0.627 | 0.531 | 1.655 | 0.099 | 0.494 | 0.622 | 1.407 | 0.161 | -0.792 | 0.429 |
| III.R | -0.098 | 0.922 | 1.832 | 0.068 | -0.133 | 0.894 | 1.345 | 0.180 | -1.468 | 0.143 |
| IV V.L | -0.389 | 0.698 | -0.478 | 0.633 | -0.957 | 0.339 | -0.711 | 0.478 | -0.194 | 0.846 |
| IV V.R | 0.596 | 0.552 | 1.202 | 0.230 | 0.210 | 0.834 | 0.972 | 0.332 | -0.817 | 0.415 |
| VI.L | 1.245 | 0.214 | -0.454 | 0.650 | -0.028 | 0.978 | -0.257 | 0.797 | 1.412 | 0.159 |
| VI.R | 0.714 | 0.476 | 1.405 | 0.161 | -0.120 | 0.905 | 1.225 | 0.222 | -0.392 | 0.696 |
| VIIB.L | 0.484 | 0.629 | 1.388 | 0.166 | 0.156 | 0.876 | 1.310 | 0.191 | -0.981 | 0.328 |
| VIIB.R | -1.281 | 0.201 | 0.741 | 0.460 | -0.775 | 0.439 | 0.285 | 0.776 | **-2.100** | **0.037** |
| VIII.L | -1.766 | 0.078 | -2.397 | 0.017 | -2.052 | 0.041 | -2.433 | 0.016 | -0.669 | 0.504 |
| VIII.R | -2.323 | 0.021 | -2.937 | 0.004 | -2.439 | 0.015 | -2.860 | 0.005 | -0.097 | 0.923 |
| IX.L | -1.020 | 0.309 | -2.118 | 0.035 | -1.352 | 0.177 | -2.081 | 0.038 | 0.758 | 0.449 |
| IX.R | -1.402 | 0.162 | -0.079 | 0.937 | -1.243 | 0.215 | -0.224 | 0.823 | -0.328 | 0.743 |
| X.L | -0.540 | 0.590 | 1.252 | 0.212 | -0.295 | 0.768 | 0.750 | 0.454 | -0.309 | 0.758 |
| X.R | 0.138 | 0.891 | 0.762 | 0.447 | 0.154 | 0.878 | 0.676 | 0.500 | -0.337 | 0.736 |
| Vermis I II | -0.982 | 0.327 | -0.349 | 0.728 | -0.821 | 0.412 | -0.678 | 0.498 | -1.742 | 0.083 |
| Vermis III | 1.680 | 0.094 | 1.230 | 0.220 | 1.500 | 0.135 | 1.142 | 0.255 | 1.131 | 0.259 |
| Vermis IV V | 0.173 | 0.863 | -0.591 | 0.555 | -0.400 | 0.689 | -0.603 | 0.547 | -0.107 | 0.915 |
| Vermis VI | 1.425 | 0.155 | 0.113 | 0.910 | 0.898 | 0.370 | 0.262 | 0.794 | **2.332** | **0.020** |
| Vermis VII | 2.017 | 0.045 | 0.539 | 0.590 | 2.290 | 0.023 | 0.836 | 0.404 | **2.043** | **0.042** |
| Vermis VIII | 0.027 | 0.978 | -0.851 | 0.396 | -0.168 | 0.867 | -0.779 | 0.437 | 1.244 | 0.214 |
| Vermis IX | -0.006 | 0.995 | -0.882 | 0.378 | -0.379 | 0.705 | -0.657 | 0.511 | 1.216 | 0.225 |
| Vermis X | -0.063 | 0.950 | 0.067 | 0.947 | 0.245 | 0.807 | 0.136 | 0.892 | -0.070 | 0.944 |
| **LMCI vs AD** | | | | | | | | | | |
| CrusI.L | -0.956 | 0.340 | 0.510 | 0.610 | -2.352 | 0.019 | 0.195 | 0.845 | **-3.796** | **<0.001** |
| CrusI.R | -0.536 | 0.592 | -0.307 | 0.759 | -2.230 | 0.027 | -0.481 | 0.631 | -1.327 | 0.186 |
| CrusII.L | -1.626 | 0.105 | 1.087 | 0.278 | -2.183 | 0.030 | 0.419 | 0.676 | **-3.830** | **<0.001** |
| CrusII.R | -0.509 | 0.611 | 1.193 | 0.234 | -1.695 | 0.091 | 0.872 | 0.384 | **-3.014** | **0.003** |
| III.L | -0.366 | 0.714 | 0.500 | 0.618 | -1.561 | 0.120 | 0.372 | 0.710 | **-2.268** | **0.024** |
| III.R | 0.222 | 0.825 | 0.519 | 0.605 | -1.200 | 0.231 | 0.528 | 0.598 | **-2.099** | **0.037** |
| IV V.L | -0.305 | 0.761 | -0.512 | 0.609 | -2.418 | 0.016 | -0.662 | 0.508 | -1.636 | 0.103 |
| IV V.R | -0.272 | 0.786 | -1.454 | 0.147 | -2.749 | 0.006 | -1.440 | 0.151 | -0.650 | 0.516 |
| VI.L | -0.679 | 0.498 | -1.161 | 0.247 | -2.786 | 0.006 | -1.242 | 0.216 | -0.241 | 0.809 |
| VI.R | -0.235 | 0.814 | -0.157 | 0.875 | -2.406 | 0.017 | -0.321 | 0.749 | -1.900 | 0.059 |
| VIIB.L | -0.207 | 0.836 | 2.437 | 0.016 | -1.054 | 0.293 | 1.914 | 0.057 | **-3.175** | **0.002** |
| VIIB.R | -0.022 | 0.982 | 1.020 | 0.309 | -1.032 | 0.303 | 0.758 | 0.449 | **-1.971** | **0.050** |
| VIII.L | -0.091 | 0.928 | 2.495 | 0.013 | -1.104 | 0.271 | 2.003 | 0.046 | **-2.418** | **0.016** |
| VIII.R | 0.474 | 0.636 | 1.956 | 0.052 | -0.994 | 0.321 | 1.631 | 0.104 | -1.824 | 0.069 |
| IX.L | -1.283 | 0.201 | 0.334 | 0.739 | -2.403 | 0.017 | 0.135 | 0.893 | **-2.733** | **0.007** |
| IX.R | -0.397 | 0.692 | 1.078 | 0.282 | -2.848 | 0.005 | 0.917 | 0.360 | -0.709 | 0.479 |
| X.L | 1.553 | 0.122 | 3.111 | 0.002 | 0.562 | 0.574 | 2.989 | 0.003 | 0.143 | 0.886 |
| X.R | 0.877 | 0.381 | 2.578 | 0.011 | -0.240 | 0.811 | 2.503 | 0.013 | -0.157 | 0.875 |
| Vermis I II | -0.342 | 0.733 | 0.698 | 0.486 | -1.035 | 0.302 | 0.601 | 0.548 | -1.911 | 0.057 |
| Vermis III | -0.786 | 0.433 | 0.513 | 0.609 | -1.894 | 0.059 | 0.217 | 0.828 | **-2.431** | **0.016** |
| Vermis IV V | 0.110 | 0.913 | -0.346 | 0.730 | -1.880 | 0.061 | -0.351 | 0.726 | -0.138 | 0.891 |
| Vermis VI | 0.483 | 0.630 | 0.348 | 0.728 | -1.181 | 0.239 | 0.226 | 0.822 | **-2.485** | **0.014** |
| Vermis VII | -0.347 | 0.729 | 1.422 | 0.156 | -1.756 | 0.080 | 1.039 | 0.300 | **-2.885** | **0.004** |
| Vermis VIII | -0.400 | 0.689 | 0.358 | 0.721 | -1.787 | 0.075 | 0.289 | 0.773 | **-2.788** | **0.006** |
| Vermis IX | 0.272 | 0.786 | -0.298 | 0.766 | -1.666 | 0.097 | -0.223 | 0.824 | -1.085 | 0.279 |
| Vermis X | 0.682 | 0.496 | 1.000 | 0.318 | -1.227 | 0.221 | 1.023 | 0.307 | -0.505 | 0.614 |

**S8 Tabel.** Performance Parameters of Five ML Models in Classifying AD Progression.

| Model | parameter |
| --- | --- |
| LR | max_iter=1000, random_state=10 |
| SVM | kernel=rbf, probability=True, random_state=10 |
| Multilayer Perceptron Classifier | 'activation': 'relu', 'alpha': 0.0001, 'hidden_layer_sizes': (50,), 'learning_rate': 'constant', 'learning_rate_init': 0.1, 'max_iter': 200, 'solver': 'adam' |
| eXtreme Gradient Boosting | 'colsample_bytree': 1.0, 'gamma': 0.1, 'learning_rate': 0.1, 'max_depth': 6, 'n_estimators': 200, 'reg_alpha': 0.1, 'reg_lambda': 1, 'subsample': 0.8 |
| RF | bootstrap=True, max_depth=38, criterion='entropy', min_samples_leaf=11, min_samples_split=9, max_features='log2', n_estimators=288,  random_state=42 |
| K-Nearest Neighbors | n_neighbors=5 |

**S9 Table** Classification performance of cerebellar graph-theoretical features for CN vs.AD, CN vs. EMCI, EMCI vs. LMCI, and LMCI vs. AD in the training and testing sets.

| **AD vs CN** | | | | | | |
| --- | --- | --- | --- | --- | --- | --- |
| Classifier | Models | AUC | Accuracy | Sensitivity | Specificity | 95% CI |
| LR | Training | 0.949 | 0.872 | 0.858 | 0.888 | (0.921, 0.973) |
|  | Testing | 0.897 | 0.809 | 0.808 | 0.810 | (0.831, 0.952) |
| SVM | Training | 0.951 | 0.858 | 0.842 | 0.878 | (0.922, 0.974) |
|  | Testing | 0.871 | 0.798 | 0.769 | 0.833 | (0.794, 0.932) |
| MLP | Training | 0.841 | 0.762 | 0.825 | 0.684 | (0.785, 0.892) |
|  | Testing | 0.732 | 0.713 | 0.769 | 0.643 | (0.620, 0.827) |
| XGBoost | Training | 0.987 | 0.922 | 0.916 | 0.929 | (0.974,0.995) |
|  | Testing | 0.950 | 0.862 | 0.887 | 0.829 | (0.904,0.983) |
| RF | Training | 0.953 | 0.872 | 0.833 | 0.918 | (0.928, 0.974) |
|  | Testing | 0.860 | 0.787 | 0.731 | 0.857 | (0.780, 0.927) |
| KNN | Training | 0.945 | 0.858 | 0.866 | 0.849 | (0.916, 0.968) |
|  | Testing | 0.801 | 0.755 | 0.793 | 0.707 | (0.711, 0.885) |
| **CN vs EMCI** | | | | | | |
| LR | Training | 0.998 | 0.983 | 0.991 | 0.975 | (0.994, 1.0) |
|  | Testing | 0.995 | 0.963 | 0.936 | 0.981 | (0.986, 1.0) |
| SVM | Training | 0.998 | 0.978 | 0.973 | 0.983 | (0.995, 1.0) |
|  | Testing | 0.981 | 0.951 | 0.915 | 0.981 | (0.950, 0.999) |
| MLP | Training | 0.999 | 0.978 | 0.982 | 0.975 | (0.997, 1.0) |
|  | Testing | 0.968 | 0.912 | 0.894 | 0.925 | (0.930, 0.993) |
| XGBoost | Training | 0.997 | 0.974 | 0.946 | 1.000 | (0.991, 1.0) |
|  | Testing | 0.971 | 0.921 | 0.936 | 0.906 | (0.938, 0.996) |
| RF | Training | 0.992 | 0.961 | 0.955 | 0.966 | (0.981, 0.998) |
|  | Testing | 0.967 | 0.915 | 0.894 | 0.906 | (0.934, 0.989) |
| KNN | Training | 0.986 | 0.935 | 0.964 | 0.908 | (0.974, 0.995) |
|  | Testing | 0.930 | 0.855 | 0.894 | 0.811 | (0.871, 0.976) |
| **EMCI vs LMCI** | | | | | | |
| LR | Training | 0.981 | 0.929 | 0.905 | 0.948 | (0.963, 0.995) |
|  | Testing | 0.964 | 0.911 | 0.913 | 0.909 | (0.925, 0.991) |
| SVM | Training | 0.988 | 0.948 | 0.916 | 0.974 | (0.976, 0.997) |
|  | Testing | 0.943 | 0.878 | 0.935 | 0.818 | (0.895, 0.981) |
| MLP | Training | 0.988 | 0.948 | 0.937 | 0.957 | (0.974, 0.998) |
|  | Testing | 0.953 | 0.867 | 0.870 | 0.864 | (0.908, 0.986) |
| XGBoost | Training | 0.983 | 0.924 | 0.863 | 0.974 | (0.969, 0.994) |
|  | Testing | 0.940 | 0.844 | 0.761 | 0.932 | (0.879, 0.983) |
| RF | Training | 0.983 | 0.914 | 0.874 | 0.948 | (0.969, 0.993) |
|  | Testing | 0.914 | 0.867 | 0.826 | 0.909 | (0.845, 0.968) |
| KNN | Training | 0.944 | 0.838 | 0.684 | 0.965 | (0.915, 0.968) |
|  | Testing | 0.855 | 0.756 | 0.587 | 0.932 | (0.775, 0.927) |
| **LMCI vs AD** | | | | | | |
| LR | Training | 0.810 | 0.745 | 0.776 | 0.714 | (0.748, 0.865) |
|  | Testing | 0.632 | 0.612 | 0.698 | 0.524 | (0.556, 0.778) |
| SVM | Training | 0.900 | 0.740 | 0.725 | 0.755 | (0.784, 0.892) |
|  | Testing | 0.605 | 0.600 | 0.605 | 0.595 | (0.539, 0.767) |
| MLP | Training | 0.639 | 0.617 | 0.694 | 0.541 | (0.560, 0.712) |
|  | Testing | 0.584 | 0.553 | 0.605 | 0.500 | (0.459, 0.708) |
| XGBoost | Training | 0.963 | 0.872 | 0.962 | 0.772 | (0.937, 0.984) |
|  | Testing | 0.631 | 0.588 | 0.703 | 0.500 | (0.506, 0.747) |
| RF | Training | 0.925 | 0.862 | 0.894 | 0.826 | (0.886, 0.957) |
|  | Testing | 0.624 | 0.600 | 0.676 | 0.542 | (0.505, 0.737) |
| KNN | Training | 0.795 | 0.725 | 0.769 | 0.674 | (0.734, 0.853) |
|  | Testing | 0.523 | 0.518 | 0.514 | 0.521 | (0.405, 0.640) |

**References**

1. Jagust WJ, Koeppe RA, Rabinovici GD, Villemagne VL, Harrison TM, Landau SM. The ADNI PET Core at 20. Alzheimer’s & Dementia. 2024;20: 7340–7349. doi:10.1002/alz.14165

2. Rahman M TP. Neuroanatomy, Pons. 2020.
